# Optimal exercise modalities and dosages for improving depression in middle-aged and older adults with Parkinson's disease: A Bayesian Dose–response network meta-analysis

**DOI:** 10.1371/journal.pone.0354206

**Published:** 2026-07-23

**Authors:** Yiang Lyu, Jiawei Chen, Siqin Zeng

**Affiliations:** 1 College of Physical Education, Hunan Agricultural University, Changsha, China; 2 Faculty of Physical Education, National Research Tomsk State University, Tomsk, Russia; 3 Hunan Traditional Chinese Medical College, Zhuzhou, Hunan, China; Mayo Clinic College of Medicine and Science, UNITED STATES OF AMERICA

## Abstract

**Objective:**

To characterise the dose response relationship between exercise and depressive symptoms in middle aged and older adults with Parkinson disease using a continuous modelling framework and to compare structural differences across exercise modalities.

**Methods:**

PubMed, Embase, Web of Science, Cochrane Library and APA PsycINFO were searched to 19 February 2026. Exercise dose was standardised as MET-minutes per week. A Bayesian network meta-regression model was used to estimate continuous dose–response relationships. Evidence certainty was assessed using GRADE.

**Results:**

Twenty-five RCTs were included. Overall exercise dose showed a significant non-linear association with reduced depressive symptoms in middle-aged and older adults with Parkinson disease. Beneficial effects were sustained between 110 and 890 MET-minutes per week, peaking at 560 MET-minutes per week (effect size 0.48, 95% credible interval 0.22–0.80), with an optimal range of 440–670 MET-minutes per week. Effects were not stable at 1000 MET-minutes per week. Modality analyses indicated that exercise combined with cognitive training and mind–body exercise demonstrated consistent benefits within moderate to moderately high dose ranges, both peaking at 560 MET-minutes per week. Aerobic cycling, functional training, and multicomponent training showed no stable significant dose range, whereas walking reached significance only at ≥670 MET-minutes per week with wide credible intervals.

**Conclusion:**

Exercise in middle-aged and older adults with Parkinson disease and comorbid depression exhibits a structured non-linear dose–response pattern with a defined optimal range, and modality-specific differences indicate that therapeutic effects depend on both quantitative dose and qualitative task characteristics, providing a quantitative basis for precision exercise prescription.

## 1 Introduction

According to projections from the Global Burden of Disease Study 2021, the global number of people living with Parkinson disease is expected to increase by 112 percent from 2021 to 2050, reaching approximately 25.2 million cases by 2050 [1]. Population ageing is identified as the dominant driver, this trajectory indicates a steadily expanding public health burden [1]. Depression is among the most prevalent neuropsychiatric manifestations of Parkinson disease in middle aged and older adults. Approximately 40–50 percent of patients experience clinically meaningful anxiety or depressive symptoms. These conditions are closely associated with faster disease progression, poorer treatment adherence, a higher comorbidity load and increased mortality risk [2,3].

Current management of Parkinson disease related depression commonly includes antidepressant medications such as selective serotonin reuptake inhibitors and tricyclic antidepressants, optimisation of dopaminergic therapy and psychological interventions including cognitive behavioural therapy, however, evidence for antidepressant efficacy in Parkinson disease populations remains inconsistent [4]. Tricyclic antidepressants may exacerbate cognitive impairment and psychiatric symptoms. Selective serotonin reuptake inhibitors may influence motor manifestations and they carry potential interaction risks when combined with monoamine oxidase B inhibitors [4]. As a result, achieving safe and sustained improvement in depressive symptoms while maintaining motor control remains a major challenge in the comprehensive management of Parkinson disease in middle aged and older adults [5].

Exercise interventions have become increasingly integrated into multidisciplinary care for Parkinson disease in middle aged and older adults. Exercise is widely regarded as a promising option for improving depressive symptoms. Positive signals have been reported across modalities including aerobic training, resistance training, tai chi and multicomponent programmes. Yet most studies have prioritised whether exercise works rather than specifying which dose is most effective [6–9]. Exercise dose encompasses intensity, frequency and duration. Dose not only shapes the magnitude of benefit but also influences adherence, safety and long term sustainability. The absence of clearly defined dose boundaries limits the development of practical exercise prescriptions. It also constrains rigorous comparisons across modalities [10]. Defining modality specific dose response characteristics for depression improvement is therefore essential for moving exercise from feasibility based recommendations to precision oriented prescribing.

Against this background, the present study synthesised evidence from RCTs to characterise dose features across exercise modalities and to examine their association with changes in depressive symptoms in middle aged and older adults with Parkinson disease. The aim was to identify potential optimal dose ranges and modality related differences. By constructing dose response models, this work seeks to provide an operational quantitative basis for exercise prescription in Parkinson disease related depression and to strengthen evidence informed individualisation in clinical practice.

## 2 Methodology

### 2.1 Information sources and search strategy

To ensure methodological rigour and transparency, the study protocol was developed in accordance with PRISMA guidance and prospectively registered on PROSPERO (CRD420261321631). We searched PubMed, Embase, Web of Science, the Cochrane Library, and APA PsycINFO from database inception to 19 February 2026. Because indexing systems and search syntaxes differ across databases, tailored search strategies were developed for each source. Controlled vocabulary terms, including MeSH and Emtree, were combined with free text keywords using Boolean operators to maximise sensitivity while limiting irrelevant retrieval. To reduce the risk of missed studies, we also performed backward citation screening of reference lists from relevant systematic reviews and meta analyses published within the past decade.

All records were deduplicated before screening. Two reviewers independently screened titles and abstracts followed by full text assessment. Disagreements were resolved through discussion. A third reviewer adjudicated when consensus could not be reached. The full search strategies and operational details are provided in S1 File 1 to support reproducibility.

### 2.2 Eligibility criteria

To enhance comparability across studies and improve coherence of evidence synthesis, eligibility criteria were defined a priori using the PICOS framework.

#### Population.

We included participants with Parkinson disease diagnosed using standard clinical criteria. The target population was middle aged and older adults. This was operationalised using the age characteristics reported in each trial, including a mean age of at least 50 years or samples described as predominantly middle aged or older. Sex and geographical region were not restricted. Studies were excluded if the sample included multiple neurological disorders and Parkinson disease specific data could not be extracted separately.

#### Intervention.

The experimental group was required to receive structured exercise training as the core intervention component. Exercise modality was not restricted and could include aerobic training, resistance training, tai chi or dance based programmes, multicomponent exercise, mind body exercise, or other structured training protocols. Eligible trials had to report prespecified exercise prescription details that were verifiable and sufficient for dose quantification and consistency checking. This included intensity or an intensity proxy that could be converted, frequency, session duration, and total intervention duration or total dose. Studies were excluded when exercise was a minor element within a multicomponent programme and its independent contribution could not be separated.

#### Comparator.

Control conditions could include usual care, health education, waiting list, maintenance of habitual lifestyle, or other non exercise comparators. Trials comparing one exercise programme with another were also eligible. This enabled network comparisons and modality specific analyses, provided that random allocation and the comparator structure were appropriate and the groups were designed to be comparable.

#### Outcomes.

The primary outcome was depression related measures. Eligible studies had to use validated rating scales or clinical assessment tools and report data that supported between group comparisons. This included baseline and follow up means and standard deviations, change scores, or other convertible statistics. Studies were excluded from the primary analyses when they reported only qualitative conclusions without computable quantitative data and additional information could not be obtained from study authors.

#### Study design.

Only RCTs were included. To focus on dose effects of sustained exercise interventions rather than acute or transient responses, we excluded acute single bout exercise studies and very short term trials. Intervention duration was required to be at least four weeks or explicitly described as long term or continuous training. This threshold was used to ensure that the included interventions represented repeated training exposure with sufficient prescription stability for weekly dose estimation, while avoiding the inclusion of brief exposure studies that were unlikely to reflect sustained exercise effects.

#### Exclusion criteria.

We excluded non original research and non peer reviewed sources, including reviews, protocols, commentaries, conference abstracts, and theses. We excluded non randomised designs, studies without a control group, and trials in which random allocation could not be verified. We excluded studies with critical missing data that prevented effect size estimation and could not be resolved through author contact, including missing means, measures of variability, or sample sizes. We excluded highly complex interventions that incorporated medication adjustment, electrical stimulation, occupational therapy, or other therapeutic components when the independent contribution of exercise could not be isolated. We also excluded studies in which device based, surgical, or implant based treatments were the primary approach and exercise was only ancillary, particularly when the exercise prescription was unclear or could not be quantified. Studies with a methodological quality score below 4 and those judged to be at high risk of bias were also excluded [11].

### 2.3 Data curation and processing

During data preparation, the study team implemented a prespecified dual independent workflow. Two reviewers independently confirmed eligibility and extracted data. Extracted information was cross checked after completion. When discrepancies arose regarding eligibility decisions or extracted variables, the reviewers first resolved them through discussion. If disagreement persisted, an additional team member who was not involved in the initial screening and extraction adjudicated. This process was designed to minimise selection bias and preserve independence of decisions.

Data were organised using a structured extraction template that captured four domains. First, bibliographic characteristics were recorded, including first author, publication year, and study setting or country. Second, sample characteristics were extracted, including group specific sample sizes, age descriptors such as mean age, range, or equivalent reporting, and sex distribution. Third, intervention characteristics and dose components were documented. This included exercise modality, training frequency, session duration, and intervention period. Information required for standardising dose across trials was also recorded. Fourth, outcome data were extracted. This included the depression assessment instrument and group level means with measures of dispersion at baseline and follow up, or alternative statistics that could be converted to effect size inputs.

When continuous outcomes were not reported as mean and standard deviation, we applied established conversion methods. Means and standard deviations were derived from the median, interquartile range, range, and sample size as appropriate to ensure consistency of effect estimation [12,13]. If a single study reported multiple scales or indicators for the same depression outcome at the same time point, only one measure was included in the primary analysis to avoid double counting participants and inflating study weight. The selection followed a prespecified hierarchy. Priority was given to the primary scale specified by the authors or to instruments with broader clinical use and stronger evidence for reliability and validity. Additional measures were reserved for sensitivity analyses or supplementary reporting when applicable.

### 2.4 Quality assessment

Methodological quality of included trials was assessed independently by two reviewers under blinded and independent conditions. Ratings were recorded and then cross checked for agreement. Discrepancies were addressed through item by item comparison against the original reports. If consensus could not be achieved, a third reviewer performed an independent review and provided the final judgement. This procedure aimed to reduce the influence of subjective interpretation on study level quality grading.

Overall trial quality was evaluated using the Physiotherapy Evidence Database scale. The scale contains 11 items. The first item relates to external validity and is not included in the total score. The remaining 10 items contribute to a summary score ranging from 0 to 10. For each scored item, 1 point was assigned when the criterion was clearly satisfied, whereas 0 points were assigned when the criterion was not satisfied or was not reported. In accordance with the prespecified protocol, trials scoring below 4 were considered to have insufficient methodological quality and were excluded from quantitative synthesis. Scores of 4–5 indicated fair quality. Scores of 6–7 indicated moderate quality. Scores of 8 or higher indicated high quality [14,15]. The Physiotherapy Evidence Database scale was selected because its criteria are clearly defined and operationally straightforward. Its 0–10 scoring approach has been widely adopted in previous systematic reviews and meta-analyses of physiotherapy, rehabilitation, and exercise intervention trials. It captures key dimensions of trial design and reporting that are central to internal validity.

### 2.5 Analytical framework and computational workflow

The primary objective of this study was to characterise dose dependent patterns of exercise related improvement in depressive symptoms among middle aged and older adults with Parkinson disease. We evaluated whether changes in exercise exposure were associated with quantifiable gains in effect. We also compared the relative performance of exercise modalities across dose ranges. To achieve these aims, we applied a Bayesian random effects network dose response model based on model based network meta analysis. This framework integrates evidence from both direct and indirect comparisons and enables inference across studies on a harmonised dose scale [16].

Before model fitting, we examined network connectivity to confirm that exercise modalities and control conditions formed a connected structure suitable for network inference. We then evaluated key elements that support the transitivity assumption. These elements included age characteristics, baseline depression severity, disease stage, and intervention duration. Comparability across treatment comparisons was assessed to ensure that effect modifiers were not unevenly distributed. We also conducted consistency checks to evaluate whether direct and indirect evidence showed systematic divergence. This step was used to confirm an appropriate basis for integrated inference [17].

Effect sizes were expressed as standardised mean differences. Small sample bias was addressed using Hedges g as the common metric. Uncertainty was summarised using ninety five percent credible intervals [18]. To support interpretation of dose information and effect direction, we first visualised trial level dose and effect estimates using scatter plots. We then specified a set of candidate dose response functions to accommodate potential non linear relationships. Candidate forms included an Emax structure, restricted cubic splines, a quadratic polynomial, and additional specifications that can represent non monotonic patterns [19]. Model selection considered goodness of fit and parsimony. Criteria included the deviance information criterion, parameter identifiability, residual behaviour, and the magnitude of between study heterogeneity. The primary model was chosen based on interpretability and consistency of posterior predictions under stable convergence. Posterior dose response curves were then generated for each exercise modality along with the corresponding posterior distributions [20].

For dose harmonisation, exercise interventions were first classified by modality according to the trial prescriptions. Exercise intensity was standardised using metabolic equivalent values derived from the Adult Compendium of Physical Activities 2024 and the Older Adult Compendium of Physical Activities [21,22]. Because no Parkinson disease-specific compendium of MET values is currently available, MET values from the 2024 Adult Compendium of Physical Activities and the Older Adult Compendium of Physical Activities were used as a standardised reference for dose harmonisation across trials. Given that the target population consisted of middle-aged and older adults with Parkinson disease, the Older Adult Compendium was used to improve age-related applicability where relevant. MET values were assigned according to the specific exercise modality reported in each original trial prescription. Weekly dose was calculated as session duration multiplied by weekly frequency to obtain weekly training time. This value was then multiplied by the assigned metabolic equivalent to derive MET minutes per week as the dose variable [23]. Because dose ranges varied substantially across studies, modelling dose as a purely continuous variable could yield unstable estimates and increase sensitivity to extreme values. We therefore applied prespecified dose stratification within 150–2000 MET minutes per week to improve comparability across studies and facilitate model convergence. Sensitivity analyses evaluated the robustness of findings to alternative stratification schemes [24]. All analyses were performed in R version 4.4.2. Network dose response modelling and posterior inference were implemented using the MBNMAdose package. Visualisations were produced with ggplot2 to display dose effect curves, credible interval bands, and modality comparisons.

### 2.6 Certainty of evidence

Certainty of evidence for key outcomes was assessed using a dual independent rating process. Two reviewers independently assigned certainty ratings according to prespecified criteria and documented the supporting rationale. When discrepancies occurred, a third team member reviewed the disputed items. Consensus was then reached through discussion to minimise bias arising from individual judgement. We evaluated certainty of evidence using the GRADE framework [25]. This approach considers risk of bias related to study design and conduct, consistency of findings across studies, directness of the evidence in relation to the target population and intervention, precision of effect estimates as reflected by interval width and information size, and the likelihood of publication bias. Each key outcome was ultimately classified into one of four categories. These categories were high, moderate, low, or very low certainty.

## 3 Results

### 3.1 Literature search and study selection

**Fig 1:**
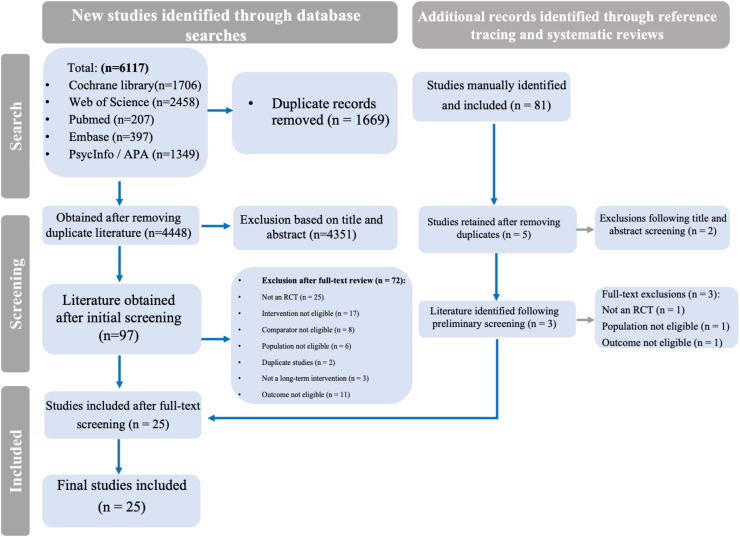
PRISMA flow diagram of the study selection process.

The systematic search was completed on 19 February 2026 and covered five databases. These were the Cochrane Library, Web of Science, PubMed, Embase, and APA PsycINFO. The search yielded 6117 records. After title and abstract screening, 97 articles proceeded to full text assessment. During full text review, studies were excluded for predefined reasons. Common reasons included interventions in which exercise was not the core component, inappropriate control conditions, populations that were not middle aged or older adults with Parkinson disease, insufficient data on depression outcomes, and study designs that were not RCTs. Twenty five trials identified through database searching met the eligibility criteria. We additionally screened reference lists from prior systematic reviews and related studies. This process identified 81 potentially relevant records. No additional eligible studies were included after applying the same screening procedures. In total, 25 RCTs were included. The selection process is summarised in Fig 1. Detailed search strategies for all databases are provided in S1 Text.

### 3.2 Characteristics of included studies

This meta analysis synthesised evidence from 25 RCTs. All participants were clinically diagnosed with Parkinson disease in middle aged and older adults, and depressive symptoms were assessed as a primary or key outcome across studies. The target population was defined consistently. Included trials focused on Parkinson disease without mixing samples from other neurological conditions. This ensured that the findings were specific to Parkinson disease related depression in middle aged and older adults. Mean age across studies clustered around 60 years. Sex distributions varied across trials. The overall age structure was comparable, which supports demographic comparability of the included populations.

Across all trials, structured exercise training was delivered as the principal intervention. Exercise modalities covered mind body programmes, functional training approaches, and multicomponent combinations. To enable harmonised dose synthesis and cross trial comparisons, exercise intensity was standardised using metabolic equivalent values. Most interventions were delivered two to three times per week. Session duration typically ranged from 30 to 60 minutes. Most interventions were delivered two to three times per week, with session duration typically ranging from 30 to 60 minutes. Exercise modalities were identified from the original intervention descriptions and linked with the corresponding MET values used for weekly dose calculation. Detailed sample characteristics, exercise modalities, prescription parameters, assigned MET values, and weekly dose calculation details are summarised in S1 Table.

### 3.3 Methodological quality and risk of bias

Quality assessment using the Physiotherapy Evidence Database scale indicated that overall methodological quality was moderate to high. Across the 25 trials, scores ranged from 4 to 9, with a mean of 6.24 and a standard deviation of 1.16. Seventeen trials scored at least 6, representing 68 percent of the included evidence base. S2 Table provides study level ratings. This distribution suggests that most trials fell within the moderate quality range. Few trials were classified as low quality, which provides an acceptable basis for subsequent evidence synthesis.

Several limitations reflect structural constraints that are common in exercise intervention research. Participant blinding and intervention provider blinding were not feasible in most trials. Outcome assessment procedures were generally better controlled. Many studies reported assessor blinding or equivalent safeguards in measurement procedures, which reduces the likelihood of detection bias. In addition, most trials described random allocation procedures and baseline comparability across groups. These features support internal validity of the pooled inferences. Overall, although design constraints were unavoidable, performance across key quality domains was broadly consistent and risks of systematic error appeared manageable.

### 3.4 Effects of exercise dose on depression in Parkinson disease in middle aged and older adults

#### 3.4.1 Network consistency and comparability of dose nodes.

Before fitting continuous dose response models, we assessed network consistency and comparability across dose levels. S3 and S4 Tables report these evaluations. The aim was to determine whether direct comparisons between dose levels, indirect comparisons derived through network pathways, and the final network estimates were compatible in both direction and magnitude. This step reduces the risk that structural inconsistency would amplify bias during subsequent modelling.

Across most dose contrasts, the credible intervals of the network estimates generally fell within the ranges covered by direct and indirect evidence. Substantial overlap was observed. No systematic reversals in direction or marked deviations were detected, suggesting that network synthesis did not introduce additional inconsistency. For key contrasts involving control conditions or typical moderate dose nodes, direct, indirect, and network estimates showed consistent interval locations and widths. This pattern supports adequate transitivity across dose nodes. In contrasts where no clear dose signal was present, intervals commonly crossed the null across all evidence sources. This further indicates that network outputs aligned with the conclusions supported by the original evidence. Taken together, these findings support the statistical assumptions required for continuous dose response modelling in the present evidence structure.

#### 3.4.2 Dose response findings for overall exercise dose.

**Fig 2:**
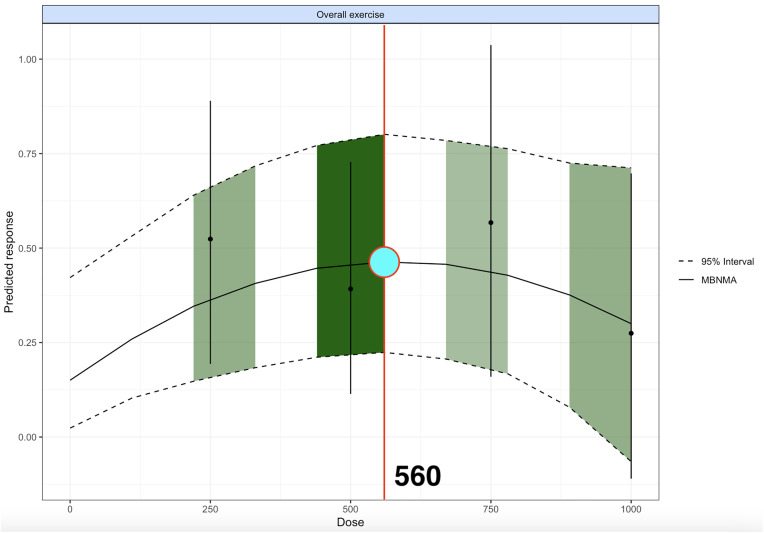
Non-linear dose–response relationship between overall exercise dose and predicted effect size. Note: The solid black curve represents the estimated dose-response relationship derived from the Bayesian model-based network meta-analysis (MBNMA). The dashed lines indicate the 95% credible interval. Black dots represent observed effect estimates at specific dose levels, with vertical lines indicating corresponding uncertainty intervals. The red vertical line marks the estimated optimal dose (560 MET-min/week), and the highlighted point indicates the predicted effect at this dose. Shaded areas represent the distribution of available evidence across dose ranges.

Posterior predictions from the Bayesian network meta regression model are presented in Fig 2, S5 Table, S1 Fig, S3 Fig and S4 Fig. When exercise dose was expressed as MET minutes per week, overall exercise showed a clear non linear dose response relationship with improvement in depressive symptoms among middle aged and older adults with Parkinson disease. Across the range of 110–890 MET minutes per week, the ninety five percent credible intervals for all dose nodes did not cross zero, indicating stable statistically significant benefits.

Effect estimates increased progressively with dose. The posterior means were 0.277 at 110 MET minutes per week, 0.361 at 220, 0.422 at 330, and 0.460 at 440. The predicted peak occurred at 560 MET minutes per week with a posterior mean of 0.477 and a ninety five percent credible interval from 0.224 to 0.801. Beyond this point, estimates declined modestly. The posterior means were 0.468 at 670, 0.436 at 780, and 0.382 at 890. These nodes remained statistically significant although the magnitude of benefit attenuated. When dose increased to 1000 MET minutes per week, the credible interval crossed zero. The posterior mean was 0.306, indicating that effects at very high dose were no longer stable. Overall, the advantageous range for overall exercise dose was concentrated in the moderately high dose band of approximately 440–670 MET minutes per week. The optimal predicted node was 560 MET minutes per week.

#### 3.4.3 Dose response findings by exercise modality.

**Fig 3:**
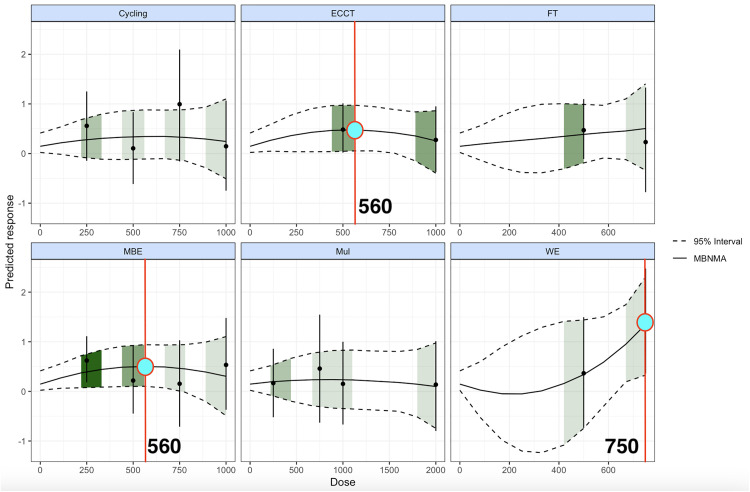
Dose–response relationships across different exercise modalities. Note: Panels display modality-specific dose-response curves estimated using the MBNMA framework. The solid line represents the predicted effect across increasing exercise dose, and dashed lines indicate the 95% credible interval. Points denote observed study-level effect estimates with uncertainty intervals. The red vertical line indicates the modality-specific optimal dose where applicable. Shaded regions illustrate the distribution and density of available evidence within each dose range. Abbreviations: ECCT, Exercise combined with cognitive training; FT, Functional training; MBE, Mind–body exercise; Mul, Multicomponent exercise; WE, Walking exercise.

Exercise modalities differed in both the shape of the dose effect curves and the ranges over which statistical significance was maintained. These results are shown in Fig 3, S6 Table, S2 Fig, S5 Fig and S6 Fig. Exercise combined with cognitive training demonstrated statistically significant benefits across 110–780 MET minutes per week, with the lower bound of the ninety five percent credible interval remaining above zero at all dose nodes. Effect estimates increased with dose and reached a predicted peak at 560 MET minutes per week with a posterior mean of 0.487. Statistical support weakened at higher doses. Mind body exercise followed a similar pattern. Significant improvements were maintained across 110–670 MET minutes per week. The maximal predicted effect also occurred at 560 MET minutes per week with a posterior mean of 0.504. Beyond this dose, credible intervals began to cross zero.

In contrast, aerobic cycling, functional training, and multicomponent training did not show a stable significant dose range. For these modalities, the ninety five percent credible intervals crossed zero at all dose nodes. Walking did not demonstrate significant improvement at low to moderate doses. Statistical significance emerged only at high dose nodes at or above 670 MET minutes per week. Credible intervals for walking at high doses were comparatively wide, indicating greater uncertainty and less stable estimation. The apparent high dose advantage for walking therefore warrants cautious interpretation within the current evidence structure. Overall, exercise combined with cognitive training and mind body exercise showed the most stable benefits within moderate to moderately high dose ranges. Other modalities did not demonstrate a consistent significant advantageous dose interval.

### 3.5 Summary certainty of evidence

A total of 25 RCTs were included. Certainty of evidence for the primary outcome of depressive symptoms was graded using the GRADE framework. Detailed judgements are reported in S7 Table. Overall certainty for the primary outcome was rated as moderate. Downgrading was driven by risk of bias and was judged as serious. No downgrading was applied for inconsistency, indirectness, or imprecision. Publication bias was not detected.

This moderate certainty rating indicates that, despite design constraints that are common in exercise intervention trials, including limited feasibility of blinding, the body of evidence showed consistent direction of effects, stable findings across analyses, and acceptable precision of estimates. Within a reasonable range of uncertainty, the available data support an association between structured exercise interventions and improvement in depressive symptoms among middle aged and older adults with Parkinson disease. The overall inference is therefore supported by a reliable interpretive basis.

### 3.6 Publication bias and small study effects

**Fig 4:**
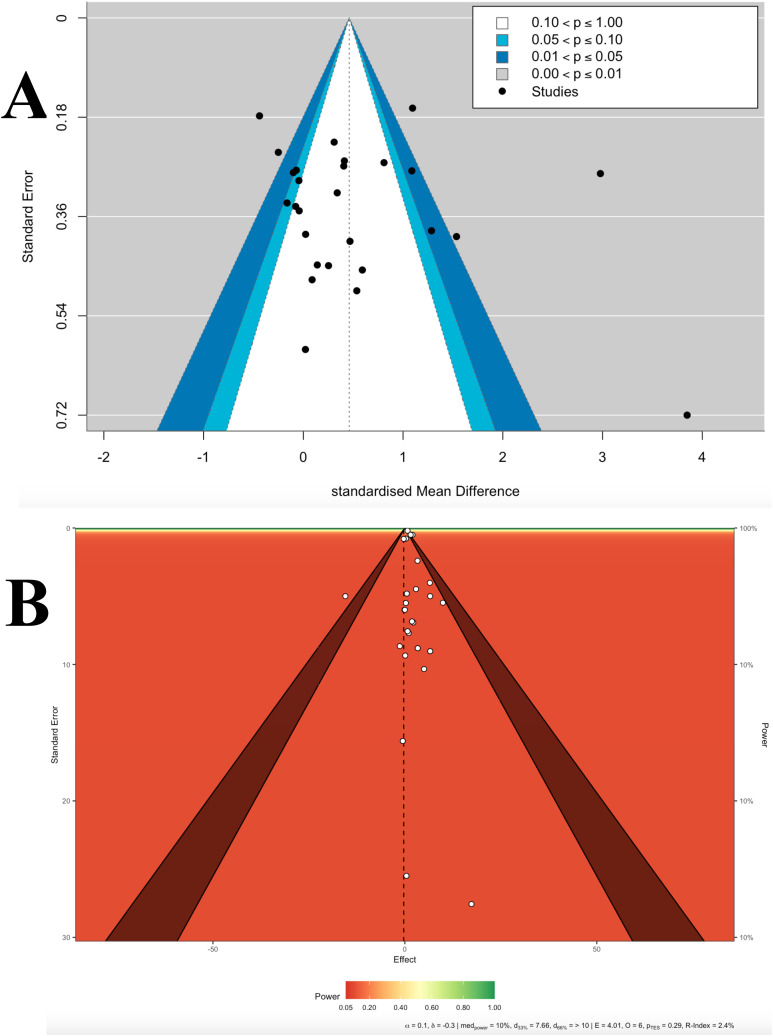
Assessment of publication bias and small-study effects. Note: Panel A presents the standard funnel plot used to assess potential publication bias. The x-axis represents the standardized mean difference (SMD), and the y-axis represents the standard error. The vertical dashed line indicates the pooled effect estimate. Shaded regions correspond to different levels of statistical significance (p-value contours). Black dots represent individual studies. Panel B shows the contour-enhanced funnel plot, which aids in distinguishing publication bias from other sources of asymmetry. The color gradient at the bottom represents statistical power distribution. The studies appear to be symmetrically distributed around the pooled effect estimate, with no evident pattern of asymmetry.

To evaluate potential small study effects and systematic publication bias, we examined funnel plot asymmetry for the pooled effect estimates. Results are shown in Fig 4. Egger regression did not indicate significant asymmetry with z equal to 1.619 and p equal to 0.105. This finding suggests that statistically detectable publication bias was not present within the current evidence structure. We additionally estimated the limit effect as the standard error approached zero. The limit estimate was 0.187 with a ninety five percent confidence interval from minus 0.161 to 0.536. The interval included the null and did not support a systematic shift in effect. Taken together, the regression test and the limit estimate did not provide clear evidence of small study effects. The pooled findings therefore appear relatively stable with respect to publication bias.

### 3.7 Robustness analyses

To examine stability and generalisability of the dose response inference, we conducted sensitivity analyses across three domains. These domains were model specification, restrictions by study quality, and influence of individual studies. First, we compared multiple candidate non linear dose effect functions. Model choice was guided by fit metrics and interpretability of parameters. A quadratic function was selected as the primary specification for inference. Second, we repeated modelling under stricter quality constraints by retaining only higher quality or more rigorously designed trials. The overall shape of the dose response curve and the advantageous dose range were consistent with the primary analysis. No reversals in effect direction were observed. Key dose nodes did not show meaningful displacement. Third, we performed leave one out analyses to assess leverage of single studies. We also monitored studies that contributed information at high doses, including those above 1500 MET minutes per week. Removal of any individual trial did not produce dominant changes in the pooled estimates or in the dose classification. Across these checks, the main conclusions remained stable. The dose response findings were robust to alternative modelling choices, variations in sample composition, and exclusion of individual studies.

## 4 Discussion

### 4.1 Key findings

This study provides the first systematic evidence in middle aged and older adults with Parkinson disease and comorbid depression that exercise dose relates to improvement in depressive symptoms through a structured pattern rather than an empirical association. Continuous dose modelling with network evidence integration indicates a clearly non linear response. The trajectory resembles a functional curve with a peak range instead of a linear accumulation of benefit. This pattern suggests threshold and saturation features in the pathways through which exercise influences depression related regulation. Within an appropriate dose range, exercise may be associated with improvements in depressive symptoms through several potential pathways described in previous literature, including reward processing, stress-responsive systems, motivation, and behavioural activation. However, these mechanisms were not directly examined in the present study [26–28]. When dose continues to increase beyond a certain level, additional physiological load and perceived strain may offset part of the benefit. Marginal gains then attenuate [29,30]. These findings provide a dose-based framework for interpreting exercise effects on Parkinson disease related depression in middle aged and older adults, while the underlying mechanisms remain to be further validated. Improvement in depressive symptoms is unlikely to be a simple function of energy expenditure. It is more consistent with a dynamic balance between neurobiological regulatory gains and the costs imposed by added load.

Differences in the shapes of modality specific dose curves further highlight the importance of exercise content for improving depressive symptoms [31]. Exercise combined with cognitive training and mind body exercise showed more stable advantages within moderate dose ranges. In contrast, training that is primarily oriented toward physical capacity did not yield a consistent advantageous dose interval. Evidence syntheses suggest that the mental health benefits of physical activity are mediated largely through affective processes, self regulation capacity, and psychosocial mechanisms rather than being determined solely by energy expenditure [32]. This supports the view that improvement in depressive symptoms may depend on deeper engagement related mechanisms beyond metabolic intensity alone. Within a multilevel mechanistic framework, physical activity can induce neuroplastic changes at molecular, structural, and functional levels. These changes appear particularly relevant to prefrontal systems involved in executive control and regulation [32,33]. It is therefore plausible that modalities requiring cognitive engagement and sustained self regulation may recruit prefrontal control systems. Such recruitment has been proposed to contribute to adaptive regulation of depression-related neuroprocesses based on previous neurobiological evidence, although this pathway was not directly assessed in the present study. This interpretation does not diminish the role of metabolic intensity. Available evidence indicates that antidepressant effects of exercise likely reflect coordinated biological and psychosocial pathways [34]. Cognitive engagement and regulatory demands may represent complementary mechanisms alongside metabolic routes. The theoretical contribution of this study is to move the field from a binary question of effectiveness toward a structural account of which dose ranges and modality characteristics are more likely to yield stable improvement in depressive symptoms. This provides a mechanistically informed framework for defining dose boundaries and refining precision exercise prescriptions for middle aged and older adults with Parkinson disease and comorbid depression.

### 4.2 Limits of dose as an explanatory construct

Although this study identified relatively stable advantageous dose ranges, a further question is whether exercise dose as a single continuous construct is sufficient to explain heterogeneity in improvement of depressive symptoms in middle aged and older adults with Parkinson disease. Prior work indicates that Parkinson disease related depression involves abnormalities in monoaminergic systems, with particular relevance to dopaminergic dysfunction. These changes are accompanied by dysregulation of cortico striatal control networks and are often linked to impairment in executive control [3,35]. Within this context, previous studies have suggested that exercise may influence depression-related regulation through modulation of neural activity in prefrontal inhibitory control circuits. One study reported that a single bout of moderate intensity exercise increased N2 and P3 amplitudes and improved inhibitory control performance. These changes coincided with immediate improvement in depressive state measures. Yet even at comparable intensity, exercise modalities can produce distinct neural and regulatory effects. Open skill exercise has been associated with broader cortical activation and larger behavioural gains [36].

These observations suggest that neuroregulatory effects of exercise are not driven solely by dose in a linear manner. They may be shaped by modality features and task structure [37]. Dose remains an important determinant of intervention effects. The optimal range may vary with individual differences in neural integrity and dopaminergic status. Neuroimaging studies have further suggested that exercise may influence cortico-striatal connectivity and dopaminergic-related markers [38,39]. This supports the possibility that dose response relationships are state dependent rather than constant across disease stages. Exercise dose provides a critical quantitative axis for understanding depression outcomes in Parkinson disease in middle aged and older adults. Its explanatory power nevertheless has structural limits. Future research should extend dose modelling by integrating modality specific characteristics, mechanistic neural indicators, and clinical phenotypes. Progress will require moving from a single variable approach to multidimensional interaction models. Such models are more likely to capture the pathways through which exercise improves depressive symptoms.

A further limitation concerns the use of standard compendium-derived MET values for dose harmonisation. Although the Adult and Older Adult Compendiums provide a transparent and reproducible framework for comparing exercise dose across studies, these MET values were not derived specifically from individuals with Parkinson disease. Motor symptoms, gait impairment, rigidity, postural instability, disease severity, and medication status may alter the actual energy cost of a given activity in this population. Therefore, MET-minutes/week in this study should be interpreted as a standardised relative dose metric for evidence synthesis rather than as an individual-level estimate of physiological energy expenditure. Future trials should report measured or population-specific energy expenditure data to refine dose estimation in Parkinson disease.

### 4.3 Study limitations and future directions

Although this study provides structured evidence through dose modelling and network integration, caution is warranted when extending the interpretation beyond the current evidence base. First, the analyses were based on secondary synthesis of published RCTs. Exercise dose was harmonised using a unified metric, yet trials differed in how interventions were organised, how closely training was supervised, and the contexts in which participants engaged with the programmes. The present models therefore primarily delineate the structural relationship between dose ranges and improvement in depressive symptoms. They do not directly test how implementation context modifies depression related regulation.

Second, depressive symptom scores were used as the primary outcome. These measures capture clinically relevant change, but they do not provide direct mechanistic evidence. Neuroimaging markers, biological measures, and indices of dopaminergic function were not integrated. As a result, although we propose a framework involving prefrontal regulation and cortico striatal network engagement based on prior literature, this study does not establish a direct chain linking dose, mechanistic indicators, and improvement in depressive symptoms. Future studies incorporating multimodal mechanistic measures, including neuroimaging, electrophysiological assessments, and biological markers, are required to validate these proposed pathways. Future work should embed multimodal mechanistic measures within dose prediction models. Longitudinal designs are needed to move from behavioural inference to mechanistic validation.

Third, the synthesis focused on overall characteristics of middle aged and older adults with Parkinson disease and comorbid depression. Subgroup heterogeneity across disease stage, cognitive status, functional capacity, and medication status was not modelled with fine granularity. Although disease severity, commonly indexed by the Hoehn and Yahr scale, and medication status may be clinically relevant to exercise response, these variables were not consistently or sufficiently reported across the included RCTs. Several studies did not provide extractable group level data suitable for meta-regression. Including these factors as moderators would therefore have substantially reduced the analysable evidence base and increased model instability. Future studies should report disease stage, medication status, baseline depressive symptom severity, and individual functional profiles in a standardised manner to support more clinically stratified dose response modelling.

In addition, modality-specific and high-dose dose–response estimates should be interpreted cautiously. The absence of a stable significant dose range for aerobic cycling and multicomponent training should not be interpreted as definitive evidence of no efficacy for depressive symptoms. Rather, these findings may reflect sparse data within certain modality-dose nodes and heterogeneity in intervention content. This limitation is particularly relevant for multicomponent training, because programmes classified under this category may combine aerobic, resistance, balance, functional, cognitive, or behavioural components in different ways. Such variability may dilute a consistent modality-specific dose signal and reduce the stability of the estimated dose–response curves. A similar caution applies to walking exercise. Although walking reached statistical significance only at higher dose nodes of 670 MET-minutes per week or above, this should not be interpreted as evidence that low-to-moderate dose walking is ineffective. The finding may reflect limited trial representation, uneven dose distribution, and wider uncertainty around walking-specific estimates at lower dose levels. Walking remains a clinically accessible and commonly recommended exercise option, but the present model indicates that, within the available evidence for depressive symptoms in Parkinson disease, a clearer statistical signal was observed only at higher weekly doses. Similarly, the instability observed at 1000 MET-minutes per week cannot be clearly distinguished as either a true biological saturation effect or an artefact of limited high-dose evidence. Given the sparse data and wider uncertainty at high-dose nodes, this finding should be interpreted primarily as reduced statistical stability rather than as definitive evidence of a harmful or ineffective high-dose threshold. Future trials with adequately represented modality-specific and high-dose prescriptions are needed to distinguish biological saturation from data-driven uncertainty and to clarify the dose range over which walking exercise may provide stable antidepressant benefits.

Overall, this study offers a dose structured framework for exercise in Parkinson disease related depression in middle aged and older adults. Its primary value lies in establishing a foundation for mechanism integrated and precision oriented research. Progress will require moving beyond single variable dose models toward multidimensional interaction models that integrate modality characteristics, mechanistic indicators, and clinical phenotypes. Such approaches are likely to support a transition from empirical optimisation to mechanism informed precision regulation [40,41].

## 5 Conclusion

This study applied a Bayesian network dose response model to establish a structured explanatory framework for exercise interventions in middle aged and older adults with Parkinson disease and comorbid depression. The findings indicate that exercise effects do not increase linearly with dose. Benefits follow a non linear pattern characterised by an advantageous range. This pattern is consistent with a dynamic balance between neuroregulatory gains and physiological load. Differences across exercise modalities further suggest that intervention effects are shaped by exercise content and task characteristics, not by dose alone. By shifting the focus from whether exercise works to which dose structures and modality combinations yield more stable benefit, this work defines quantitative boundaries and a conceptual framework for refining exercise based strategies in this population. It also provides a foundation for future precision modelling that incorporates individual state, mechanistic indicators, and modality specific features.

## Supporting information

S1 Text Search strategies and terms across electronic databases. Detailed search terms, strings, and combinations used for populations, interventions, and outcomes across Cochrane Library, Web of Science, PubMed, Embase, and PsycInfo/APA databases.
(DOCX)

S1 Table Characteristics of the included studies, exercise modalities, prescription parameters, assigned MET values, and weekly exercise dose calculation. Complete extraction data for the 25 included randomized controlled trials, encompassing study design, sample size, gender, age, exercise modality, prescription parameters (weeks, frequency, duration), assigned metabolic equivalents (METs), control conditions, and depression outcome measures.
(DOCX)

S2 Table Methodological quality and risk of bias assessment via the PEDro scale. Item-by-item breakdown and total consensus scores (out of 10) for all 25 included trials across the eleven specific criteria of the Physiotherapy Evidence Database scale.
(DOCX)

S3 Table Node-splitting analysis of inconsistency for the overall exercise dose model. Direct, indirect, and network (MBNMA) effect estimates with their corresponding 95% credible intervals and Bayesian p-values for overall exercise dose comparisons against baseline.
(DOCX)

S4 Table Node-splitting analysis of inconsistency across specific exercise modalities. Inconsistency evaluations, direct and indirect comparative evidence, and combined model-based estimates for distinct exercise types at varying dose levels.
(DOCX)

S5 Table Posterior dose–response estimates for overall exercise versus placebo. Detailed posterior distribution metrics, including means, standard deviations, and specific quantiles (0.025, 0.25, 0.5, 0.75, and 0.975), modeled across standardized overall exercise doses (110–1000 MET-minutes/week).
(DOCX)

S6 Table Predicted dose–response effects by individual exercise modality and dose. Comprehensive posterior probability parameters and interval distributions across multiple exercise types (Cycling, ECCT, FT, MBE, Mul, WE), stratified by cumulative dose levels.
(DOCX)

S7 Table Certainty of evidence assessment using the GRADE framework. Detailed evaluation of the quality of evidence for the primary depressive symptom outcome, including justifications for downgrading domains and the final moderate certainty rating.
(DOCX)

S1 Fig Network geometry of overall exercise across standardized dose levels. Structural network plot where nodes represent intervention conditions categorized by exercise dose levels and edges signify direct head-to-head trial comparisons. Node size is proportional to sample capacity, and line weight reflects trial frequency.
(DOCX)

S2 Fig Network geometry of distinct exercise modalities across different dose levels. Circular network map illustrating the comparative data structure among various exercise modalities (Cycling, ECCT, FT, MBE, Mul, WE) at specific dose values relative to the placebo reference node.
(DOCX)

S3 Fig Distribution of posterior mean effects across overall exercise dose categories. Boxplots displaying the distribution, median values, interquartile ranges, and outlier profiles of modeled posterior responses across standardized exercise dosages.
(DOCX)

S4 Fig Individual dose–response trajectories of overall exercise effects on the link scale. Scatter-and-line plots mapping individual estimated response trajectories and variance patterns across cumulative exercise doses.
(DOCX)

S5 Fig Distribution of posterior mean effects across exercise modalities and dose levels. Panel-specific boxplots tracking modality-dependent median effect estimates, distributions, and variance metrics across cumulative dose levels.
(DOCX)

S6 Fig Dose–response patterns across specific exercise modalities on the link scale. Modality-stratified trajectory charts representing individual and modeled posterior response trends across cumulative standardized dose ranges.
(DOCX)

S1 File PRISMA 2020 checklist. The completed checklist indicates the page(s) of the manuscript on which each PRISMA 2020 reporting item is addressed.
(DOCX)
