# Supplementary material for: Optimal exercise modalities and dosages for improving depression in middle-aged and older adults with Parkinson's disease: A Bayesian Dose–response network meta-analysis
Source: PLoS One. 2026 Jul 23;21(7):e0354206. doi: 10.1371/journal.pone.0354206 (PMC13395444; doi:10.1371/journal.pone.0354206)
Supplement: S1 Text — Detailed search terms, strings, and combinations used for populations, interventions, and outcomes across Cochrane Library, Web of Science, PubMed, Embase, and PsycInfo/APA databases. (DOCX) [file pone.0354206.s001.docx]

| S1 Text. Search strategies and terms across electronic databases. | | | |
| --- | --- | --- | --- |
| **Database** | **Search Terms for Population** | **Search Terms for Intervention** | **Search Terms for Outcomes** |
| Cochrane Library (limited to trials, N=1706) | Parkinsonian OR Parkinsonism OR Parkinson: ti | movement OR hydrotherapy OR sport OR locomotion OR sprint OR yoga OR activity OR kinesiotherapy OR virtual reality OR stability OR train OR swim OR strength OR exercise OR balance training OR resistance OR endurance OR motor control OR run OR mind-body exercise OR tai chi OR dance OR high intensity interval OR aerobic OR pilates OR posture OR martial art OR sports combined training OR conditioning OR stretch OR activities OR core control OR water sports OR athletic OR physical OR functional training OR isometric OR walk OR neuromuscular: ti, ab, kw | depression OR depressive OR mood disorder OR affective disorder OR mental health OR anxiety OR anxious OR psychological distress OR emotional distress: ti, ab, kw |
| Web of Science (limited to Article, N = 2458) | TI=(Parkinsonian OR Parkinsonism OR Parkinson) | AB=(movement OR hydrotherapy OR sport OR locomotion OR sprint OR yoga OR activity OR kinesiotherapy OR virtual reality OR stability OR train OR swim OR strength OR exercise OR balance training OR resistance OR endurance OR motor control OR run OR mind-body exercise OR tai chi OR dance OR high intensity interval OR aerobic OR pilates OR posture OR martial art OR sports combined training OR conditioning OR stretch OR activities OR core control OR water sports OR athletic OR physical OR functional training OR isometric OR walk OR neuromuscular) | AB=(depression OR depressive OR mood disorder OR affective disorder OR mental health OR anxiety OR anxious OR psychological distress OR emotional distress) |
| PubMed (limited to RCTs OR Clinical Trial, N = 207) | Parkinsonian[Title] OR Parkinsonism[Title] OR Parkinson[Title] | movement[Title/Abstract] OR hydrotherapy[Title/Abstract] OR sport[Title/Abstract] OR locomotion[Title/Abstract] OR sprint[Title/Abstract] OR yoga[Title/Abstract] OR activity[Title/Abstract] OR kinesiotherapy[Title/Abstract] OR virtual reality[Title/Abstract] OR stability[Title/Abstract] OR train[Title/Abstract] OR swim[Title/Abstract] OR strength[Title/Abstract] OR exercise[Title/Abstract] OR balance training[Title/Abstract] OR resistance[Title/Abstract] OR endurance[Title/Abstract] OR motor control[Title/Abstract] OR run[Title/Abstract] OR mind-body exercise[Title/Abstract] OR tai chi[Title/Abstract] OR dance[Title/Abstract] OR high intensity interval[Title/Abstract] OR aerobic[Title/Abstract] OR pilates[Title/Abstract] OR posture[Title/Abstract] OR martial art[Title/Abstract] OR sports combined training[Title/Abstract] OR conditioning[Title/Abstract] OR stretch[Title/Abstract] OR activities[Title/Abstract] OR core control[Title/Abstract] OR water sports[Title/Abstract] OR athletic[Title/Abstract] OR physical[Title/Abstract] OR functional training[Title/Abstract] OR isometric[Title/Abstract] OR walk[Title/Abstract] OR neuromuscular[Title/Abstract] | depression[Title/Abstract] OR depressive[Title/Abstract] OR mood disorder[Title/Abstract] OR affective disorder[Title/Abstract] OR mental health[Title/Abstract] OR anxiety[Title/Abstract] OR anxious[Title/Abstract] OR psychological distress[Title/Abstract] OR emotional distress[Title/Abstract] |
| Embase (limited to RCTs, N = 397) | parkinsonian:ti OR parkinsonism:ti OR parkinson:ti | movement:ab,ti OR hydrotherapy:ab,ti OR sport:ab,ti OR locomotion:ab,ti OR sprint:ab,ti OR yoga:ab,ti OR activity:ab,ti OR kinesiotherapy:ab,ti OR 'virtual reality':ab,ti OR stability:ab,ti OR train:ab,ti OR swim:ab,ti OR strength:ab,ti OR exercise:ab,ti OR 'balance training':ab,ti OR resistance:ab,ti OR endurance:ab,ti OR 'motor control':ab,ti OR run:ab,ti OR 'mind-body exercise':ab,ti OR 'tai chi':ab,ti OR dance:ab,ti OR 'high intensity interval':ab,ti OR aerobic:ab,ti OR pilates:ab,ti OR posture:ab,ti OR 'martial art':ab,ti OR 'sports combined training':ab,ti OR conditioning:ab,ti OR stretch:ab,ti OR activities:ab,ti OR 'core control':ab,ti OR 'water sports':ab,ti OR athletic:ab,ti OR physical:ab,ti OR 'functional training':ab,ti OR isometric:ab,ti OR walk:ab,ti OR neuromuscular:ab,ti | depression:ab,ti OR depressive:ab,ti OR 'mood disorder':ab,ti OR 'affective disorder':ab,ti OR 'mental health':ab,ti OR anxiety:ab,ti OR anxious:ab,ti OR 'psychological distress':ab,ti OR 'emotional distress':ab,ti |
| PsycInfo / APA (N = 1349) | Title: Parkinsonian OR Title: Parkinsonism OR Title: Parkinson | Abstract: movement OR Abstract: hydrotherapy OR Abstract: sport OR Abstract: locomotion OR Abstract: sprint OR Abstract: yoga OR Abstract: activity OR Abstract: kinesiotherapy OR Abstract: virtual reality OR Abstract: stability OR Abstract: train OR Abstract: swim OR Abstract: strength OR Abstract: exercise OR Abstract: balance training OR Abstract: resistance OR Abstract: endurance OR Abstract: motor control OR Abstract: run OR Abstract: mind-body exercise OR Abstract: tai chi OR Abstract: dance OR Abstract: high intensity interval OR Abstract: aerobic OR Abstract: pilates OR Abstract: posture OR Abstract: martial art OR Abstract: sports combined training OR Abstract: conditioning OR Abstract: stretch OR Abstract: activities OR Abstract: core control OR Abstract: water sports OR Abstract: athletic OR Abstract: physical OR Abstract: functional training OR Abstract: isometric OR Abstract: walk OR Abstract: neuromuscular: ti, ab, kw | Abstract: depression OR Abstract: depressive OR Abstract: mood disorder OR Abstract: affective disorder OR Abstract: mental health OR Abstract: anxiety OR Abstract: anxious OR Abstract: psychological distress OR Abstract: emotional distress |
