# Supplementary material for: Optimal exercise modalities and dosages for improving depression in middle-aged and older adults with Parkinson's disease: A Bayesian Dose–response network meta-analysis
Source: PLoS One. 2026 Jul 23;21(7):e0354206. doi: 10.1371/journal.pone.0354206 (PMC13395444; doi:10.1371/journal.pone.0354206)
Supplement: S1 Table — Complete extraction data for the 25 included randomized controlled trials, encompassing study design, sample size, gender, age, exercise modality, prescription parameters (weeks, frequency, duration), assigned metabolic equivalents (METs), control conditions, and depression outcome measures. (DOCX) [file pone.0354206.s002.docx]

Table S1. Characteristics of the included studies, exercise modalities, prescription parameters, assigned MET values, and weekly exercise dose calculation.

| **Source** | **Study design** | **Sample size** | **Gender (M, F)** | **Age, Mean±SD** | **Intervention type（E）** | **Weeks/Frequency per week/Duration-min** | **METs (min)/Code** | **Program（C）** | **Outcome** |
| --- | --- | --- | --- | --- | --- | --- | --- | --- | --- |
| Hiroko Hashimoto 2015 | R-T | 15/17/14 | 3,12/2,15/7,7 | 67.9±7.0/62.7±14.9/69.7±4.0 | Dance/CHPT | 12/1/60 | 6.3/0303160, 5.0/0206260 | COT | Self-rating Depression Scale |
| Silvia Rios Romenets 2015 | R-T | 18/15 | 7,8/12,6 | 64.3±8.1/63.2±9.9 | Dance | 12/2/60 | 6.3/0303160 | COT | BDI |
| Lori J.P. Altmann 2016 | R-Th-S | 11/9/10 | NA | 62.8±8.6/63.3±7.3/67.8±9.8 | CHPT/BE | 16/3/45 | 5.0/0206260, 3.5/0211360 | COT | BDI |
| Alessandro Picelli 2016 | R-T-S | 9/8 | 5,4/4,4 | 71.2±9.2/71.6±7.2 | WE | 4/3/45 | 4.5/1717060 | COT | BDI |
| Corjena Cheung 2018 | R-T-S | 10/10 | NA | 63.5±8.5/65.8±6.6 | Yoga | 12/2/60 | 2.5/0219060 | COT | BDI |
| Maria Grazia Maggio 2018 | R-T-D | 10/10 | 6,4/4,6 | 69.9±6.3/68.9±10.05 | VR | 8/3/60 | 5.5/2240060 | CT | GDS |
| Hye Gyeong Son 2018 | R-T | 33/30 | 14,19/9,21 | >60 | ECCT | 4/1/120 | 5.0/0206260 | COT | GDS |
| Kristi Michels 2018 | R-T | 9/4 | NA | 66.44/75.50 | Dance | 10/NA/60 | 6.3/0303160 | COT | BDI |
| Van der Kolk 2018 | R-T-S | 22/15 | NA | 75 | CHPT | 27/7/60 | 5.0/0206260 | COT | HADS |
| Matthew A. Sacheli 2019 | R-T-S | 20/15 | 13,7/9,6 | 66.76±5.98/67.85±8.50 | Cycling | 12/3/60 | 5.3/0101060 | COT | BDI |
| Nicolien M van der Kolk 2019 | R-T-D | 65/65 | 23,42/27,38 | 59 | Cycling | 4/3/30 | 5.3/0101060 | COT | HADS |
| Paolo Solla 2019 | R-T-S | 16/17 | 6,10/7,10 | 67.8±5.9/67.1±6.3 | Dance | 12/2/90 | 6.3/0303160 | COT | BDI |
| Farzin Hajebrahimi 2022 | R-T-S | 11/13 | 9,2/10,3 | 66.36±8.04/65.53±9.93 | VR | 4/3/60 | 5.5/2240060 | COT | GDS |
| Tsai-Chin Cheng 2022 | R-Th-S | 13/11/16 | 7,6/6,5/11,5 | 71.4±8.5/71.6±5.1/73.9±6.9 | VR | 2/5/40 | 5.5/2240060 | CT /COT | BDI |
| Jojo Yan Yan Kwok 2023 | R-T-S | 35/33 | 10,23/19,16 | 62.7±7.7/66.1±8.9 | RT | 8/NA/90 | 3.0/0205160 | CT | HADS |
| Cheng-Liang Chang 2024 | R-Th-S | 14/16/13 | 6,7/5,9/7,9 | 63.15±7.95/64.43±7.37/66.31±6.54 | Cycling/TC | 12/NA/30 | 5.3/0101060, 3.8/221060 | COT | BDI |
| Yueying Fang 2024 | R-T | 32/28 | 18,14/15,13 | 67.14±7.35/66.78±7.56 | RT | 12/NA/NA | 3.0/0205160 | COT | BDI |
| Yuanjiao Yan 2024 | R-T-S | 23/23 | 15,8/17,6 | 70.5±5.4/68.2±5.8 | CHPT | 12/3/60 | 5.0/0206260 | COT | GDS |
| Chia-Liang Tsai 2025 | R-Th | 20/21/20 | 12,8/13,8/11,9 | 65.05±6.80/64.67±8.05/67.00±7.07 | Cycling/TC | 12/NA/30 | 5.3/0101060, 3.8/221060 | COT | BDI |
| Asunción Mayoral-Moreno 2025 | R-T | 11/11 | 16,6 | 55-61 | WE | 8/3/60 | 4.5/1717060 | COT | BDI |
| Jojo Yan Yan Kwok 2025 | R-Th-S | 53/54 | 28,24/20,34 | 66.9±7.9/63.3±7.5 | Yoga | 8/1/90 | 2.5/0219060 | COT | HADS |
| Huimei Yin 2025 | R-T-S | 25/26 | 12, 13/12, 14 | 58.80±8.61/58.15±7.09 | TC | 12/5/30 | 3.8/221060 | FT | HADS |
| Costa 2025 | RCT | 11/7/10/9 | 6,5 / 5,2 / 6,4 / 6,3 | 61±8/61±9/58±9/68±13 | RT/Cycling | 8/3/40 | 3.0/0205160, 5.3/0101060 | COT | GDS |
| Jéssica A. Moratelli 2025 | RCT | 11/8/9 | NA | 63.3±8.9 | FT/ Pilates | 12/2/60 | 5/0206260, 2.5/0219060 | COT | BDI |
| Ding 2026 | RCT | 86/86 | 52, 34/57, 29 | 67.00±4.04/67.10±4.03 | ECCT | 12/3/30 | 5.0/0206260 | COT | BDI |

Notes: NA, not available; E, experimental group; C, control group; R, randomized; S, Single blind; T, Two arm; Th, Three arm; D, Double blind; COT, conventional treatment; CT, Cognitive training; BDI, Beck Depression Inventory; GDS, Geriatric Depression Scale; HADS, Hospital Anxiety and Depression Scale; TC, Tai Chi; ECCT, Exercise combined with cognitive training; VR, Virtual reality training; CHPT, Comprehensive physical training; BE, Balancing exercise; WE, Walking exercise; RT, Resistance training; Weekly exercise dose was calculated as MET-minutes/week using the following formula: MET value × session duration (minutes) × weekly frequency. For intervention arms involving more than one exercise component, MET values were assigned according to the specific exercise type reported in the original trial prescription.

Included studies:^[1-25]^

ADDIN EN.REFLIST [1] HASHIMOTO H, TAKABATAKE S, MIYAGUCHI H, et al. Effects of dance on motor functions, cognitive functions, and mental symptoms of Parkinson's disease: A quasi-randomized pilot trial [J]. Complementary Therapies in Medicine, 2015, 23(2): 210-9.<https://doi.org/10.1016/j.ctim.2015.01.010>

[2] ROMENETS S R, ANANG J, FERESHTEHNEJAD S M, et al. Tango for treatment of motor and non-motor manifestations in Parkinson's disease: A randomized control study [J]. Complementary Therapies in Medicine, 2015, 23(2): 175-84.<https://doi.org/10.1016/j.ctim.2015.01.015>

[3] ALTMANN L J P, STEGEMÖLLER E, HAZAMY A A, et al. Aerobic Exercise Improves Mood, Cognition, and Language Function in Parkinson's Disease: Results of a Controlled Study [J]. Journal of the International Neuropsychological Society, 2016, 22(9): 878-89.<https://doi.org/10.1017/s135561771600076x>

[4] PICELLI A, VARALTA V, MELOTTI C, et al. Effects of treadmill training on cognitive and motor features of patients with mild to moderate Parkinson's disease: a pilot, single-blind, randomized controlled trial [J]. Functional Neurology, 2016, 31(1): 25.

[5] CHEUNG C, BHIMANI R, WYMAN J, et al. Effects of yoga on oxidative stress, motor function, and non-motor symptoms in Parkinson’s disease: a pilot randomized controlled trial [J]. Pilot and Feasibility Studies, 2018, 4.<https://doi.org/10.1186/s40814-018-0355-8>

[6] GYEONG S H, OK C E. The Effects of Mindfulness Meditation-based Complex Exercise Program on Motor and Non-Motor Symptoms, and Quality of Life in Patients with Parkinson's Disease [J]. Asian Nursing Research, 2018: S197613171730511X-.

[7] MAGGIO M G, DE COLA M C, LATELLA D, et al. What About the Role of Virtual Reality in Parkinson Disease's Cognitive Rehabilitation? Preliminary Findings From a Randomized Clinical Trial [J]. Journal of Geriatric Psychiatry and Neurology, 2018, 31(6): 312-8.<https://doi.org/10.1177/0891988718807973>

[8] MICHELS K, DUBAZ O, HORNTHAL E, et al. "Dance Therapy" as a psychotherapeutic movement intervention in Parkinson's disease [J]. Complementary Therapies in Medicine, 2018, 40: 248-52.<https://doi.org/10.1016/j.ctim.2018.07.005>

[9] VAN DER KOLK N M, DE VRIES N M, PENKO A L, et al. A remotely supervised home-based aerobic exercise programme is feasible for patients with Parkinson's disease: results of a small randomised feasibility trial [J]. Journal of Neurology Neurosurgery and Psychiatry, 2018, 89(9): 1003-+.<https://doi.org/10.1136/jnnp-2017-315728>

[10] SACHELI M A, NEVA J L, LAKHANI B, et al. Exercise increases caudate dopamine release and ventral striatal activation in Parkinson's disease [J]. Movement Disorders, 2019, 34(12): 1891-900.<https://doi.org/10.1002/mds.27865>

[11] SOLLA P, CUGUSI L, BERTOLI M, et al. Sardinian Folk Dance for Individuals with Parkinson's Disease: A Randomized Controlled Pilot Trial [J]. Journal of Alternative and Complementary Medicine, 2019, 25(3): 305-16.<https://doi.org/10.1089/acm.2018.0413>

[12] VAN DER KOLK N M, DE VRIES N M, KESSELS R P C, et al. Effectiveness of home-based and remotely supervised aerobic exercise in Parkinson's disease: a double-blind, randomised controlled trial [J]. Lancet Neurology, 2019, 18(11): 998-1008.<https://doi.org/10.1016/s1474-4422(19)30285-6>

[13] CHENG T C, HUANG S F, WU S Y, et al. Integration of Virtual Reality into Transcranial Magnetic Stimulation Improves Cognitive Function in Patients with Parkinson's Disease with Cognitive Impairment: A Proof-of-Concept Study [J]. Journal of Parkinsons Disease, 2022, 12(2): 723-36.<https://doi.org/10.3233/jpd-212978>

[14] HAJEBRAHIMI F, VELIOGLU H A, BAYRAKTAROGLU Z, et al. Clinical evaluation and resting state fMRI analysis of virtual reality based training in Parkinson's disease through a randomized controlled trial [J]. Scientific Reports, 2022, 12(1).<https://doi.org/10.1038/s41598-022-12061-3>

[15] KWOK J Y Y, CHOI E P H, WONG J Y H, et al. A randomized clinical trial of mindfulness meditation versus exercise in Parkinson's disease during social unrest [J]. Npj Parkinsons Disease, 2023, 9(1).<https://doi.org/10.1038/s41531-023-00452-w>

[16] CHANG C L, PAN C Y, WANG T C, et al. Distinct effects of long-term Tai Chi Chuan and aerobic exercise interventions on motor and neurocognitive performance in early-stage Parkinson's disease: a randomized controlled trial [J]. European Journal of Physical and Rehabilitation Medicine, 2024, 60(4): 621-33.<https://doi.org/10.23736/s1973-9087.24.08166-8>

[17] FANG Y Y, WANG J, NIE D L, et al. Efficacy of exercise interventions combined with Selegiline in ameliorating freezing of gait in Parkinson's disease patients [J]. American Journal of Translational Research, 2024, 16(7): 2852-63.<https://doi.org/10.62347/jtrh2408>

[18] YAN Y J, XU Y F, WANG X X, et al. The effect of multi-component exercise intervention in older people with Parkinson's disease and mild cognitive impairment: A randomized controlled study [J]. Geriatric Nursing, 2024, 60: 137-45.<https://doi.org/10.1016/j.gerinurse.2024.08.028>

[19] COSTA V, MENACHO M, DA ROCHA T F, et al. Upper Limb Exercises Reduce Non-motor Symptoms and Increase Cognitive Function in Parkinson's Disease: Randomized Controlled Trial [J]. Journal of Geriatric Psychiatry and Neurology, 2025.<https://doi.org/10.1177/08919887251409414>

[20] KWOK J Y Y, CHAN L M L, LAI C A, et al. Effects of Meditation and Yoga on Anxiety, Depression and Chronic Inflammation in Patients with Parkinson's Disease: A Randomized Clinical Trial [J]. Psychotherapy and Psychosomatics, 2025, 94(2): 101-18.<https://doi.org/10.1159/000543457>

[21] MAYORAL-MORENO A, RODRÍGUEZ-SANTOS L, CHIMPÉN-LÓPEZ C A, et al. Effects of Square Stepping Exercise on Physical, Cognitive and Psychosocial Fitness in People with Parkinson's Disease [J]. Psychiatric Quarterly, 2025.<https://doi.org/10.1007/s11126-025-10197-9>

[22] MORATELLI J A, CORRÊA C L, ANDRADE A, et al. Functional training and Mat Pilates have a positive effect on non-motor symptoms improving cognition, depressive symptoms, anxiety, and happiness in people with Parkinson's disease: a randomized controlled clinical trial with follow-up Physical exercise in the non-motor symptoms of Parkinson's disease [J]. Aging & Mental Health, 2025, 29(10): 1892-901.<https://doi.org/10.1080/13607863.2025.2496728>

[23] TSAI C L, CHIEN C Y, PAN C Y, et al. Effects of long-term Tai Chi vs. aerobic exercise on antioxidant activity and cognitive function in individuals with Parkinson's disease [J]. Behavioural Brain Research, 2025, 476.<https://doi.org/10.1016/j.bbr.2024.115274>

[24] YIN H M, CHENG O M, ZHANG X, et al. Effects of Liuzijue Qigong on respiratory function among patients with Parkinson's disease: a randomized clinical trial [J]. Bmc Complementary Medicine and Therapies, 2025, 25(1).<https://doi.org/10.1186/s12906-025-04773-6>

[25] DING T, ZHANG J, JIANG X, et al. Effects of Combined Psychological and Functional Exercise Interventions on Emotion, Life Quality, and Brain-Derived Neurotrophic Factor Levels in Patients With Parkinson's Disease: A Randomized Controlled Trial [J]. International Journal of Psychiatry in Medicine, 2026.<https://doi.org/10.1177/00912174261422307>
