# Supplementary material for: Optimal exercise modalities and dosages for improving depression in middle-aged and older adults with Parkinson's disease: A Bayesian Dose–response network meta-analysis
Source: PLoS One. 2026 Jul 23;21(7):e0354206. doi: 10.1371/journal.pone.0354206 (PMC13395444; doi:10.1371/journal.pone.0354206)
Supplement: S2 Table — Item-by-item breakdown and total consensus scores (out of 10) for all 25 included trials across the eleven specific criteria of the Physiotherapy Evidence Database scale. (DOCX) [file pone.0354206.s003.docx]

Table S2. PEDro scale quality assessment of included studies

| Study | A1 | A2 | A3 | A4 | A5 | A6 | A7 | A8 | A9 | A10 | A11 | Total score (out of 10) |
| --- | --- | --- | --- | --- | --- | --- | --- | --- | --- | --- | --- | --- |
| Hiroko Hashimoto 2015 | 1 | 1 | 0 | 1 | 0 | 0 | 0 | 1 | 0 | 1 | 1 | 5 |
| Silvia Rios Romenets 2015 | 1 | 1 | 0 | 1 | 0 | 0 | 0 | 1 | 1 | 1 | 1 | 6 |
| Lori J.P. Altmann 2016 | 1 | 1 | 0 | 1 | 0 | 1 | 0 | 1 | 0 | 1 | 1 | 6 |
| Alessandro Picelli 2016 | 1 | 1 | 1 | 1 | 0 | 0 | 1 | 1 | 1 | 1 | 1 | 8 |
| Corjena Cheung 2018 | 1 | 1 | 1 | 1 | 0 | 0 | 1 | 1 | 0 | 1 | 1 | 7 |
| Maria Grazia Maggio 2018 | 1 | 1 | 0 | 1 | 1 | 0 | 1 | 1 | 0 | 1 | 1 | 7 |
| Hye Gyeong Son 2018 | 1 | 1 | 0 | 1 | 0 | 0 | 0 | 1 | 0 | 1 | 1 | 5 |
| Kristi Michels 2018 | 1 | 1 | 0 | 1 | 0 | 0 | 0 | 1 | 0 | 1 | 1 | 5 |
| Van der Kolk 2018 | 1 | 1 | 0 | 1 | 0 | 0 | 1 | 1 | 0 | 1 | 1 | 6 |
| Matthew A. Sacheli 2019 | 1 | 1 | 0 | 1 | 0 | 0 | 1 | 1 | 0 | 1 | 1 | 6 |
| Nicolien M van der Kolk 2019 | 1 | 1 | 1 | 1 | 0 | 1 | 1 | 1 | 1 | 1 | 1 | 9 |
| Paolo Solla 2019 | 1 | 1 | 0 | 1 | 0 | 0 | 1 | 1 | 0 | 1 | 1 | 6 |
| Farzin Hajebrahimi 2022 | 1 | 1 | 0 | 1 | 0 | 1 | 0 | 1 | 0 | 1 | 1 | 6 |
| Tsai-Chin Cheng 2022 | 1 | 1 | 1 | 1 | 0 | 0 | 1 | 1 | 0 | 1 | 1 | 7 |
| Jojo Yan Yan Kwok 2023 | 1 | 1 | 1 | 1 | 0 | 0 | 1 | 1 | 1 | 1 | 1 | 8 |
| Cheng-Liang Chang 2024 | 1 | 1 | 0 | 1 | 0 | 0 | 1 | 1 | 0 | 1 | 1 | 6 |
| Yueying Fang 2024 | 1 | 1 | 0 | 1 | 0 | 0 | 0 | 1 | 0 | 1 | 1 | 5 |
| Yuanjiao Yan 2024 | 1 | 1 | 0 | 1 | 0 | 0 | 1 | 1 | 1 | 1 | 1 | 7 |
| Chia-Liang Tsai 2025 | 1 | 1 | 0 | 1 | 0 | 0 | 0 | 1 | 0 | 1 | 1 | 5 |
| Asunción Mayoral-Moreno 2025 | 1 | 1 | 0 | 1 | 0 | 0 | 0 | 1 | 1 | 1 | 1 | 6 |
| Jojo Yan Yan Kwok 2025 | 1 | 1 | 1 | 1 | 0 | 0 | 1 | 0 | 1 | 1 | 1 | 7 |
| Huimei Yin 2025 | 1 | 1 | 0 | 1 | 0 | 0 | 1 | 0 | 0 | 1 | 1 | 5 |
| Costa 2025 | 1 | 1 | 1 | 1 | 0 | 0 | 1 | 0 | 1 | 1 | 1 | 7 |
| Jéssica A. Moratelli 2025 | 1 | 1 | 0 | 1 | 0 | 0 | 0 | 0 | 0 | 1 | 1 | 4 |
| Ding 2026 | 1 | 1 | 1 | 1 | 0 | 0 | 1 | 1 | 0 | 1 | 1 | 7 |

Notes: A1, participant selection criteria specified; A2, random assignment of participants to groups; A3, hidden assignment; A4, groups were similar at baseline; A5, all participants were blind; A6, all therapists were blind; A7, all assessors were blind; A8, measurement of at least one of the main outcomes was obtained from more than 85% of the participants. A9, intention-to-treat analysis was conducted; A10, results of statistical comparisons between groups for at least one main outcome were reported; A11, the study reported point and variability measures for at least one main outcome; 1, the entry is satisfied; 0, the entry is not satisfied.
