# Supplementary material for: Optimal exercise modalities and dosages for improving depression in middle-aged and older adults with Parkinson's disease: A Bayesian Dose–response network meta-analysis
Source: PLoS One. 2026 Jul 23;21(7):e0354206. doi: 10.1371/journal.pone.0354206 (PMC13395444; doi:10.1371/journal.pone.0354206)
Supplement: S3 Table — Direct, indirect, and network (MBNMA) effect estimates with their corresponding 95% credible intervals and Bayesian p-values for overall exercise dose comparisons against baseline. (DOCX) [file pone.0354206.s004.docx]

Table S3. Node-splitting analysis of inconsistency (Overall exercise)

| Comparison | p-value | Direct ([95% CrI]) | Indirect ([95% CrI]) | MBNMA ([95% CrI]) |
| --- | --- | --- | --- | --- |
| Overall exercise_1000 vs Overall exercise_500 | 0.249 | -0.004 (-1.018, 1.004) | 0.163 (0.028, 0.303) | 0.159 (0.034, 0.289) |
| Overall exercise_750 vs Overall exercise_500 | 0.229 | 0.024 (-0.557, 0.619) | 0.082 (0.016, 0.153) | 0.079 (0.017, 0.144) |
| Overall exercise_250 vs Placebo_0 | 0.069 | 0.376 (0.084, 0.683) | 0.066 (0.005, 0.131) | 0.079 (0.017, 0.144) |
