# Supplementary material for: Optimal exercise modalities and dosages for improving depression in middle-aged and older adults with Parkinson's disease: A Bayesian Dose–response network meta-analysis
Source: PLoS One. 2026 Jul 23;21(7):e0354206. doi: 10.1371/journal.pone.0354206 (PMC13395444; doi:10.1371/journal.pone.0354206)
Supplement: S4 Table — Inconsistency evaluations, direct and indirect comparative evidence, and combined model-based estimates for distinct exercise types at varying dose levels. (DOCX) [file pone.0354206.s005.docx]

Table S4. Node-splitting analysis of inconsistency

| Comparison | p-value | Direct ([95% CrI]) | Indirect ([95% CrI]) | MBNMA ([95% CrI]) |
| --- | --- | --- | --- | --- |
| MBE_500 vs FT_750 | 0.863 | 0.006 (-0.663, 0.652) | -0.112 (-0.896, 0.640) | -0.049 (-0.580, 0.439) |
| FT_500 vs ECCT_1000 | 0.714 | -0.066 (-1.068, 0.933) | 0.124 (-0.503, 0.705) | 0.099 (-0.408, 0.635) |
| MBE_250 vs Cycling_250 | 0.533 | 0.029 (-0.488, 0.540) | 0.068 (-0.118, 0.242) | 0.068 (-0.116, 0.240) |
| WE_750 vs Placebo_0 | 0.453 | 1.174 (0.144, 2.075) | 0.294 (-1.117, 1.758) | 0.979 (0.219, 1.765) |
| WE_500 vs Placebo_0 | 0.488 | 0.198 (-0.821, 1.200) | 0.774 (0.114, 1.414) | 0.653 (0.146, 1.177) |
| Mul_2000 vs Placebo_0 | 0.752 | -0.017 (-0.767, 0.769) | 0.150 (-1.101, 1.337) | 0.024 (-0.585, 0.623) |
| Mul_1000 vs Placebo_0 | 0.699 | -0.015 (-0.757, 0.666) | 0.006 (-0.362, 0.355) | 0.012 (-0.293, 0.311) |
| Mul_750 vs Placebo_0 | 0.434 | 0.202 (-0.695, 1.143) | -0.008 (-0.267, 0.256) | 0.009 (-0.219, 0.234) |
| Mul_250 vs Placebo_0 | 0.269 | 0.047 (-0.512, 0.590) | 0.001 (-0.081, 0.079) | 0.003 (-0.073, 0.078) |
| MBE_1000 vs Placebo_0 | 0.851 | 0.383 (-0.382, 1.172) | 0.460 (-0.151, 1.079) | 0.434 (-0.051, 0.904) |
| MBE_750 vs Placebo_0 | 0.437 | 0.011 (-0.809, 0.755) | 0.436 (0.001, 0.829) | 0.326 (-0.038, 0.678) |
| MBE_500 vs Placebo_0 | 0.556 | 0.089 (-0.514, 0.672) | 0.242 (-0.021, 0.500) | 0.217 (-0.025, 0.452) |
| ECCT_500 vs Placebo_0 | 0.391 | 0.305 (-0.045, 0.709) | 0.049 (-0.240, 0.323) | 0.143 (-0.069, 0.358) |
| Cycling_1000 vs Placebo_0 | 0.619 | -0.027 (-0.830, 0.763) | 0.354 (-0.396, 1.247) | 0.161 (-0.367, 0.720) |
| Cycling_750 vs Placebo_0 | 0.203 | 0.857 (-0.153, 1.819) | -0.012 (-0.444, 0.401) | 0.121 (-0.275, 0.540) |
| Cycling_500 vs Placebo_0 | 0.56 | -0.072 (-0.607, 0.513) | 0.143 (-0.168, 0.451) | 0.080 (-0.184, 0.360) |
