# Supplementary material for: Optimal exercise modalities and dosages for improving depression in middle-aged and older adults with Parkinson's disease: A Bayesian Dose–response network meta-analysis
Source: PLoS One. 2026 Jul 23;21(7):e0354206. doi: 10.1371/journal.pone.0354206 (PMC13395444; doi:10.1371/journal.pone.0354206)
Supplement: S5 Table — Detailed posterior distribution metrics, including means, standard deviations, and specific quantiles (0.025, 0.25, 0.5, 0.75, and 0.975), modeled across standardized overall exercise doses (110–1000 MET-minutes/week). (DOCX) [file pone.0354206.s006.docx]

Table S5. Posterior Dose–Response Estimates for Overall Exercise Compared with Placebo

| **agent** | **dose** | **mean** | **sd** | **0.025** | **0.25** | **0.5** | **0.75** | **0.975** |
| --- | --- | --- | --- | --- | --- | --- | --- | --- |
| Placebo | 0 | 0.1707107 | 0.1057568 | 0.02361345 | 0.08833859 | 0.1503441 | 0.2356994 | 0.4219636 |
| Overall exercise | 110 | 0.27701 | 0.1135833 | 0.10301322 | 0.19090594 | 0.2591513 | 0.3470423 | 0.5317761 |
| Overall exercise | 220 | 0.3607082 | 0.1276297 | 0.14755574 | 0.26782789 | 0.3459737 | 0.4393422 | 0.6397691 |
| Overall exercise | 330 | 0.4218052 | 0.1394485 | 0.18348977 | 0.32405848 | 0.4065104 | 0.510532 | 0.7173677 |
| Overall exercise | 440 | 0.4603011 | 0.1461085 | 0.21125835 | 0.35633887 | 0.4467127 | 0.5517523 | 0.7716487 |
| Overall exercise | 560 | 0.4765201 | 0.1478064 | 0.22387056 | 0.37189288 | 0.463198 | 0.567837 | 0.8009723 |
| Overall exercise | 670 | 0.467759 | 0.1472978 | 0.20640683 | 0.36477521 | 0.457173 | 0.559514 | 0.7847814 |
| Overall exercise | 780 | 0.4363968 | 0.1506425 | 0.16736191 | 0.32928637 | 0.4281677 | 0.5304452 | 0.7629822 |
| Overall exercise | 890 | 0.3824335 | 0.1655692 | 0.07892822 | 0.26746024 | 0.3760689 | 0.4908456 | 0.7250456 |
| Overall exercise | 1000 | 0.305869 | 0.1982764 | -0.06511258 | 0.17319579 | 0.2999765 | 0.4392596 | 0.7122807 |
