# Supplementary material for: Optimal exercise modalities and dosages for improving depression in middle-aged and older adults with Parkinson's disease: A Bayesian Dose–response network meta-analysis
Source: PLoS One. 2026 Jul 23;21(7):e0354206. doi: 10.1371/journal.pone.0354206 (PMC13395444; doi:10.1371/journal.pone.0354206)
Supplement: S6 Table — Comprehensive posterior probability parameters and interval distributions across multiple exercise types (Cycling, ECCT, FT, MBE, Mul, WE), stratified by cumulative dose levels. (DOCX) [file pone.0354206.s007.docx]

Table S6. Predicted Dose–Response Effects by Exercise Modality and Dose

| **agent** | **dose** | **mean** | **sd** | **0.025** | **0.25** | **0.5** | **0.75** | **0.975** |
| --- | --- | --- | --- | --- | --- | --- | --- | --- |
| Placebo | 0 | 0.16470176 | 0.1027054 | 0.022259772 | 0.08465013 | 0.146283838 | 0.2288234 | 0.4130462 |
| Cycling | 110 | 0.23036373 | 0.1411152 | -0.015068013 | 0.132522 | 0.215687498 | 0.3216305 | 0.5366669 |
| Cycling | 220 | 0.28237799 | 0.1945081 | -0.07481317 | 0.14936635 | 0.267825284 | 0.4080892 | 0.6827909 |
| Cycling | 330 | 0.32074453 | 0.2319186 | -0.114535673 | 0.16419198 | 0.305642809 | 0.4677841 | 0.7949562 |
| Cycling | 440 | 0.34546336 | 0.2496881 | -0.119998228 | 0.17810843 | 0.329211248 | 0.5028028 | 0.8575689 |
| Cycling | 560 | 0.35686418 | 0.2509773 | -0.110218896 | 0.18580273 | 0.342711785 | 0.5134837 | 0.8794933 |
| Cycling | 670 | 0.35304686 | 0.2469644 | -0.101287156 | 0.18081841 | 0.344798848 | 0.5088776 | 0.8753008 |
| Cycling | 780 | 0.33558183 | 0.2588939 | -0.146421341 | 0.15165134 | 0.324998467 | 0.5004391 | 0.8842411 |
| Cycling | 890 | 0.30446907 | 0.3106512 | -0.28537225 | 0.08581977 | 0.292960177 | 0.5071802 | 0.9340854 |
| Cycling | 1000 | 0.2597086 | 0.412226 | -0.507996855 | -0.02362582 | 0.243520914 | 0.5331546 | 1.1002278 |
| ECCT | 110 | 0.28243931 | 0.1368313 | 0.047950482 | 0.1878195 | 0.266400324 | 0.3653346 | 0.5867259 |
| ECCT | 220 | 0.3735786 | 0.1849097 | 0.03684863 | 0.24835128 | 0.360712181 | 0.4878891 | 0.7718591 |
| ECCT | 330 | 0.43811962 | 0.2192342 | 0.036221244 | 0.28968148 | 0.42715161 | 0.5730932 | 0.9042685 |
| ECCT | 440 | 0.47606237 | 0.2354089 | 0.038425317 | 0.3173486 | 0.466255449 | 0.6223114 | 0.9671162 |
| ECCT | 560 | 0.48711925 | 0.2345055 | 0.053213921 | 0.32746316 | 0.477594502 | 0.6296256 | 0.9752052 |
| ECCT | 670 | 0.46944744 | 0.2242891 | 0.054296111 | 0.32100263 | 0.454819017 | 0.6061623 | 0.9429729 |
| ECCT | 780 | 0.42517737 | 0.2213257 | 0.005422037 | 0.28298664 | 0.415883057 | 0.5686288 | 0.8844179 |
| ECCT | 890 | 0.35430903 | 0.2496977 | -0.148344343 | 0.19740483 | 0.354582889 | 0.5145026 | 0.8391811 |
| ECCT | 1000 | 0.25684241 | 0.3250918 | -0.394257787 | 0.05701314 | 0.255926326 | 0.4750123 | 0.8624576 |
| FT | 83 | 0.19799685 | 0.177086 | -0.13417705 | 0.08073919 | 0.19132061 | 0.308329 | 0.5604514 |
| FT | 170 | 0.23435906 | 0.2708885 | -0.29030534 | 0.05973506 | 0.233006243 | 0.4033195 | 0.7662562 |
| FT | 250 | 0.26911669 | 0.325499 | -0.382644581 | 0.05936942 | 0.26696285 | 0.4706792 | 0.9154617 |
| FT | 330 | 0.30514007 | 0.346325 | -0.387862029 | 0.0818447 | 0.302591488 | 0.5199207 | 0.9930149 |
| FT | 420 | 0.34717935 | 0.3327428 | -0.30678349 | 0.12862753 | 0.345794883 | 0.5509338 | 1.0054595 |
| FT | 500 | 0.38589244 | 0.2981887 | -0.188730525 | 0.19168061 | 0.386494301 | 0.5704973 | 0.9901543 |
| FT | 580 | 0.42587129 | 0.2697833 | -0.089078812 | 0.24690923 | 0.421899038 | 0.5984798 | 0.97163 |
| FT | 670 | 0.47236045 | 0.3102901 | -0.121159747 | 0.27235026 | 0.455214423 | 0.6730888 | 1.1007463 |
| FT | 750 | 0.51502901 | 0.4429687 | -0.337931236 | 0.22744922 | 0.504297621 | 0.803851 | 1.4038456 |
| MBE | 110 | 0.2838529 | 0.1279643 | 0.062609294 | 0.19462473 | 0.271123422 | 0.3662946 | 0.5558982 |
| MBE | 220 | 0.37738252 | 0.1655359 | 0.073267486 | 0.26702011 | 0.372970971 | 0.4841658 | 0.7191589 |
| MBE | 330 | 0.44529063 | 0.1927855 | 0.085717236 | 0.31837964 | 0.442603704 | 0.5697382 | 0.8342564 |
| MBE | 440 | 0.48757721 | 0.2068597 | 0.096709468 | 0.35233216 | 0.487294247 | 0.6215059 | 0.9085502 |
| MBE | 560 | 0.5044868 | 0.212456 | 0.103050832 | 0.36104116 | 0.500631158 | 0.6431636 | 0.9402335 |
| MBE | 670 | 0.49320112 | 0.2205026 | 0.070174079 | 0.34112687 | 0.492079834 | 0.6370641 | 0.934523 |
| MBE | 780 | 0.45629392 | 0.248511 | -0.01765614 | 0.28761596 | 0.453055897 | 0.6222839 | 0.9417341 |
| MBE | 890 | 0.3937652 | 0.3103983 | -0.202893199 | 0.1856528 | 0.390991361 | 0.5948063 | 0.9914521 |
| MBE | 1000 | 0.30561496 | 0.4096068 | -0.490710545 | 0.03467676 | 0.304263901 | 0.5773278 | 1.1038824 |
| Mul | 220 | 0.19816731 | 0.15289 | -0.077128185 | 0.09414046 | 0.191971134 | 0.2957194 | 0.5175386 |
| Mul | 440 | 0.22163125 | 0.2213343 | -0.197312738 | 0.07405644 | 0.218335547 | 0.3652307 | 0.6657307 |
| Mul | 670 | 0.23546788 | 0.2720624 | -0.290259172 | 0.05429538 | 0.234098265 | 0.4146663 | 0.7723898 |
| Mul | 890 | 0.238474 | 0.2968472 | -0.337029802 | 0.04385793 | 0.238058863 | 0.4319277 | 0.8228849 |
| Mul | 1100 | 0.23201346 | 0.3018452 | -0.354761319 | 0.03489111 | 0.230698542 | 0.4318378 | 0.8324542 |
| Mul | 1300 | 0.21738815 | 0.2968435 | -0.371788369 | 0.0241681 | 0.211502777 | 0.4101254 | 0.815056 |
| Mul | 1600 | 0.17995181 | 0.3025293 | -0.400962071 | -0.01743024 | 0.179327245 | 0.3693001 | 0.8035163 |
| Mul | 1800 | 0.14466203 | 0.344069 | -0.515751733 | -0.07951761 | 0.144523696 | 0.3609567 | 0.8448731 |
| Mul | 2000 | 0.10110645 | 0.4312649 | -0.746112714 | -0.18550141 | 0.098061565 | 0.3749501 | 0.9760678 |
| WE | 83 | 0.0273974 | 0.2825339 | -0.528809828 | -0.16024523 | 0.025103132 | 0.2181335 | 0.5995918 |
| WE | 170 | -0.04505681 | 0.4735201 | -0.981699721 | -0.34608991 | -0.048097868 | 0.2735991 | 0.8964463 |
| WE | 250 | -0.0471241 | 0.5869851 | -1.202067772 | -0.42668899 | -0.050806873 | 0.3446257 | 1.117399 |
| WE | 330 | 0.01265989 | 0.6387117 | -1.235663299 | -0.4120871 | 0.009592581 | 0.4419951 | 1.2886261 |
| WE | 420 | 0.15384848 | 0.6257565 | -1.082002433 | -0.26719594 | 0.158785528 | 0.5783583 | 1.4103247 |
| WE | 500 | 0.34506644 | 0.5584362 | -0.744308072 | -0.0345107 | 0.338674378 | 0.7252252 | 1.4385426 |
| WE | 580 | 0.59813567 | 0.459732 | -0.300484534 | 0.27794502 | 0.588020465 | 0.9190907 | 1.5015218 |
| WE | 670 | 0.95677017 | 0.3962611 | 0.192702574 | 0.69419966 | 0.95034114 | 1.2206685 | 1.7460337 |
| WE | 750 | 1.34127337 | 0.5152584 | 0.328146603 | 0.99403194 | 1.349094275 | 1.6885284 | 2.307293 |
