# Supplementary material for: Optimal exercise modalities and dosages for improving depression in middle-aged and older adults with Parkinson's disease: A Bayesian Dose–response network meta-analysis
Source: PLoS One. 2026 Jul 23;21(7):e0354206. doi: 10.1371/journal.pone.0354206 (PMC13395444; doi:10.1371/journal.pone.0354206)
Supplement: S7 Table — Detailed evaluation of the quality of evidence for the primary depressive symptom outcome, including justifications for downgrading domains and the final moderate certainty rating. (DOCX) [file pone.0354206.s008.docx]

Table S7. Certainty of Evidence Assessment Using the GRADE Approach

| Outcomes | Studies (design) | Risk of bias | Inconsistency | Indirectness | Imprecision | Publication bias | Overall certainty (GRADE) |
| --- | --- | --- | --- | --- | --- | --- | --- |
| Depressive symptoms (primary outcome) | 25 (RCTs) | Serious | Not serious* | Not serious | Not serious* | Undetected* | ⊕⊕⊕◯ MODERATE |

Notes: The certainty of evidence was assessed using the GRADE framework. Although all included studies were randomized controlled trials, the evidence was downgraded by one level for risk of bias due to recurrent methodological limitations, particularly insufficient allocation concealment, limited blinding procedures, and inconsistent application of intention‐to‐treat analyses across trials. No further downgrading was applied for inconsistency, indirectness, imprecision, or publication bias, as the direction of effects was generally coherent, the populations, interventions, and outcomes were directly aligned with the review question, and no clear indications of substantial uncertainty or reporting bias were identified. Consequently, the overall certainty of evidence was rated as moderate.
