# Supplementary material for: Optimal exercise modalities and dosages for improving depression in middle-aged and older adults with Parkinson's disease: A Bayesian Dose–response network meta-analysis
Source: PLoS One. 2026 Jul 23;21(7):e0354206. doi: 10.1371/journal.pone.0354206 (PMC13395444; doi:10.1371/journal.pone.0354206)
Supplement: S1 Fig — Structural network plot where nodes represent intervention conditions categorized by exercise dose levels and edges signify direct head-to-head trial comparisons. Node size is proportional to sample capacity, and line weight reflects trial frequency. (DOCX) [file pone.0354206.s009.docx]

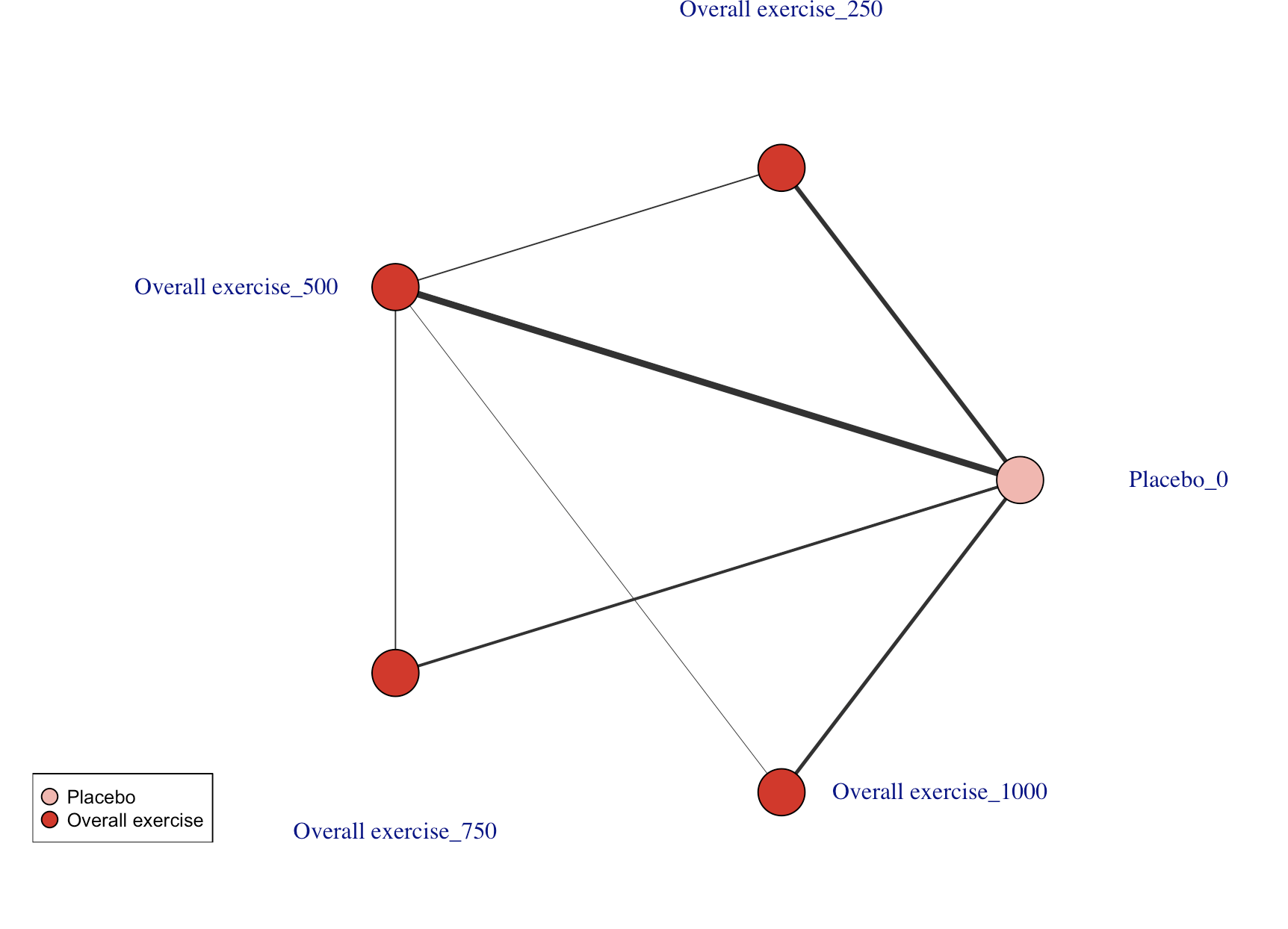


Figure S1. Network Geometry of Overall Exercise Across Dose Levels

Notes: Nodes represent intervention conditions categorized by standardized overall exercise dose levels, with node size proportional to the number of participants; edges represent direct comparisons between interventions, and edge thickness corresponds to the number of studies informing each comparison. Placebo was used as the reference comparator, and dose values indicate standardized exercise dose levels applied in the dose–response modeling.
