# Supplementary material for: Optimal exercise modalities and dosages for improving depression in middle-aged and older adults with Parkinson's disease: A Bayesian Dose–response network meta-analysis
Source: PLoS One. 2026 Jul 23;21(7):e0354206. doi: 10.1371/journal.pone.0354206 (PMC13395444; doi:10.1371/journal.pone.0354206)
Supplement: S2 Fig — Circular network map illustrating the comparative data structure among various exercise modalities (Cycling, ECCT, FT, MBE, Mul, WE) at specific dose values relative to the placebo reference node. (DOCX) [file pone.0354206.s010.docx]

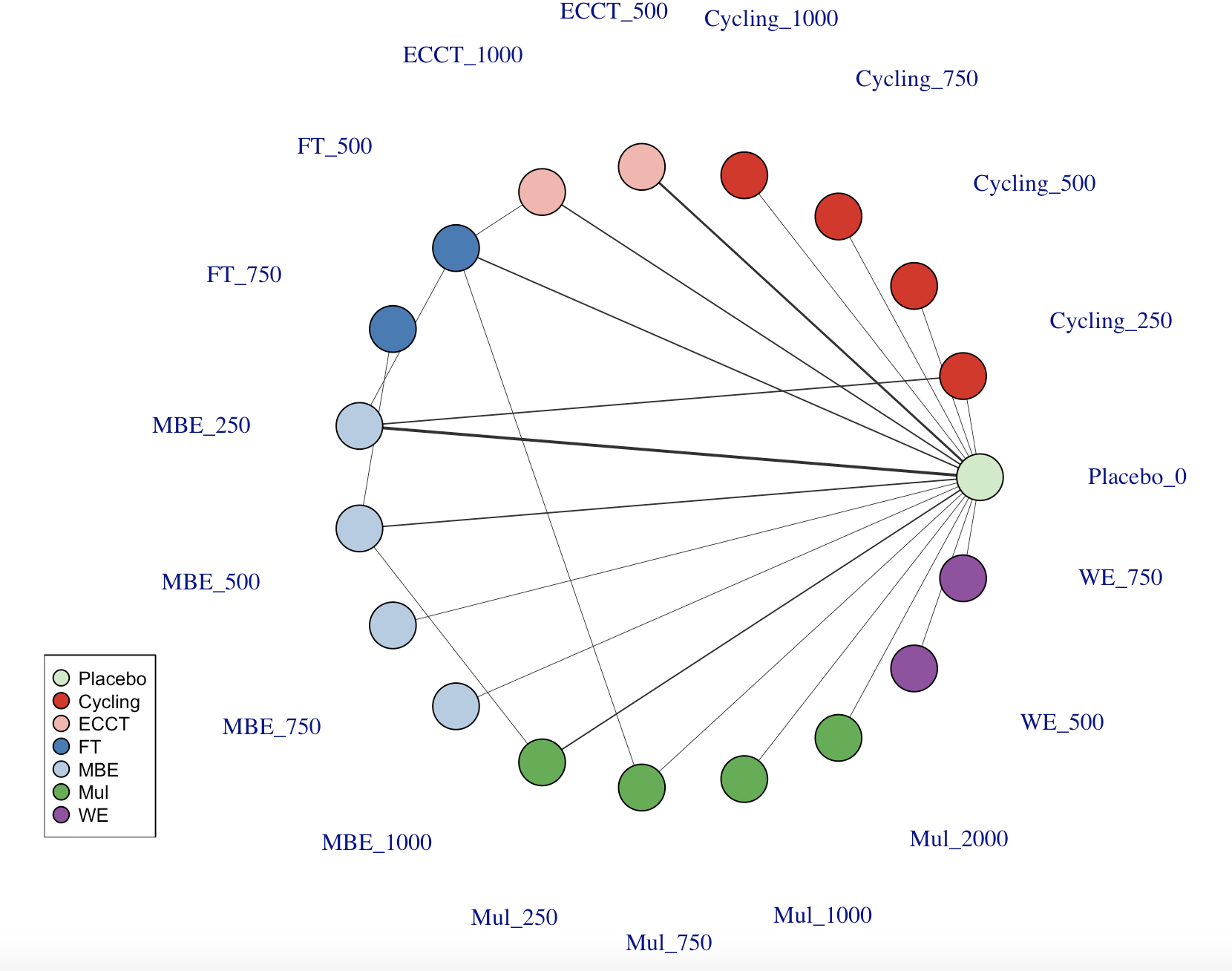


Figure S2. Network Geometry of Exercise Modalities Across Different Dose Levels

Notes: Nodes represent intervention conditions defined by exercise modality and dose level, with node size proportional to the number of participants; edges indicate direct comparisons between interventions, and edge thickness reflects the number of studies contributing to each comparison. Placebo served as the reference comparator. Abbreviations: Cycling, cycling exercise; ECCT, exercise combined with cognitive training; FT, functional training; MBE, mind-body exercise; Mul, multicomponent exercise; WE, walking exercise. Dose values indicate standardized exercise dose levels used in the dose–response modeling.
