# Supplementary material for: Optimal exercise modalities and dosages for improving depression in middle-aged and older adults with Parkinson's disease: A Bayesian Dose–response network meta-analysis
Source: PLoS One. 2026 Jul 23;21(7):e0354206. doi: 10.1371/journal.pone.0354206 (PMC13395444; doi:10.1371/journal.pone.0354206)
Supplement: S3 Fig — Boxplots displaying the distribution, median values, interquartile ranges, and outlier profiles of modeled posterior responses across standardized exercise dosages. (DOCX) [file pone.0354206.s011.docx]

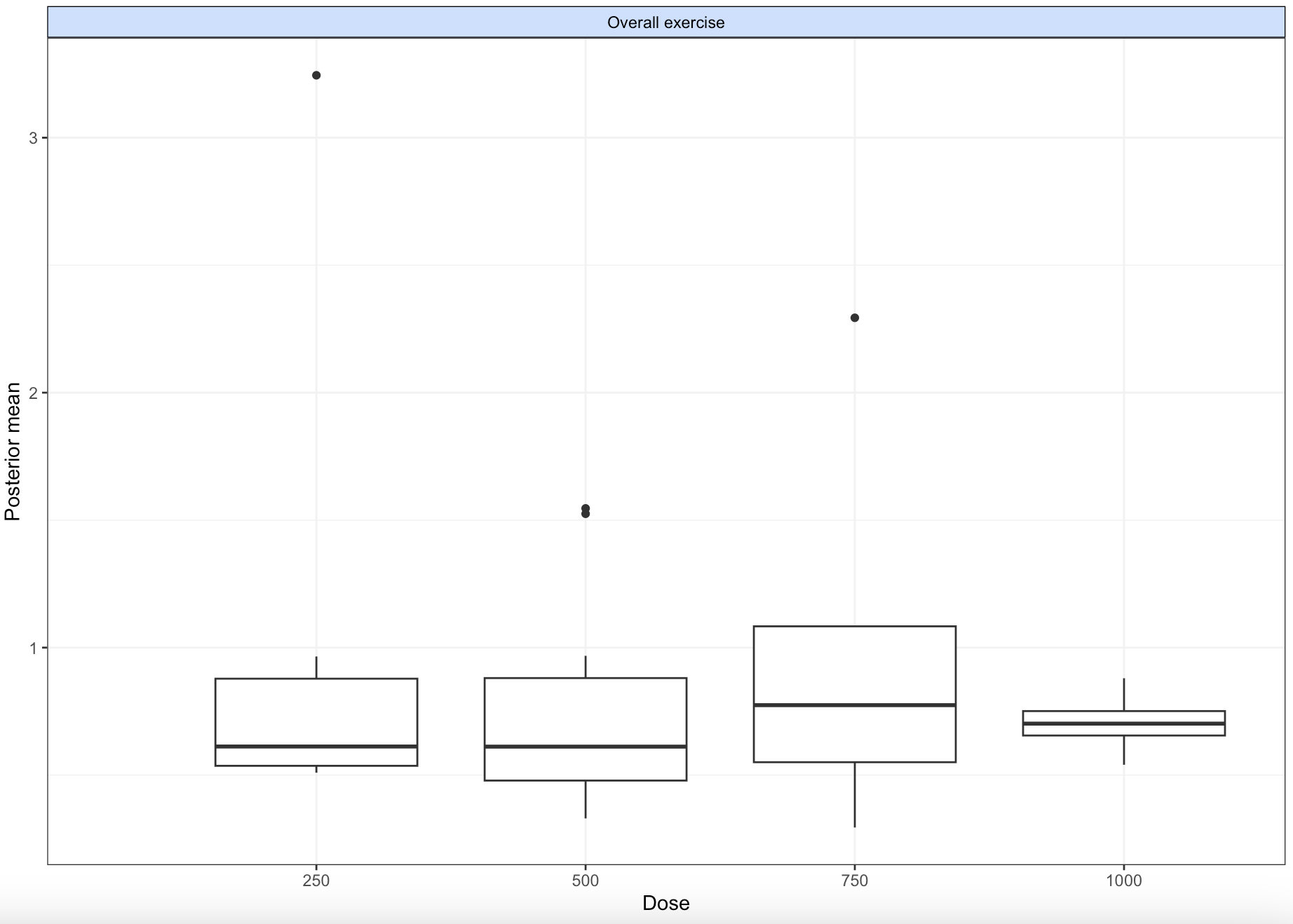


Figure S3. Distribution of Posterior Mean Effects Across Overall Exercise Dose Levels

Notes: Boxplots illustrate the distribution of posterior mean effect estimates across standardized overall exercise dose levels. Median values and interquartile ranges are displayed for each dose category, with points representing potential outliers. Overall, effect estimates showed variation across dose levels, indicating heterogeneity in posterior responses while maintaining a generally stable distribution pattern across the dose range.
