# Supplementary material for: Optimal exercise modalities and dosages for improving depression in middle-aged and older adults with Parkinson's disease: A Bayesian Dose–response network meta-analysis
Source: PLoS One. 2026 Jul 23;21(7):e0354206. doi: 10.1371/journal.pone.0354206 (PMC13395444; doi:10.1371/journal.pone.0354206)
Supplement: S4 Fig — Scatter-and-line plots mapping individual estimated response trajectories and variance patterns across cumulative exercise doses. (DOCX) [file pone.0354206.s012.docx]

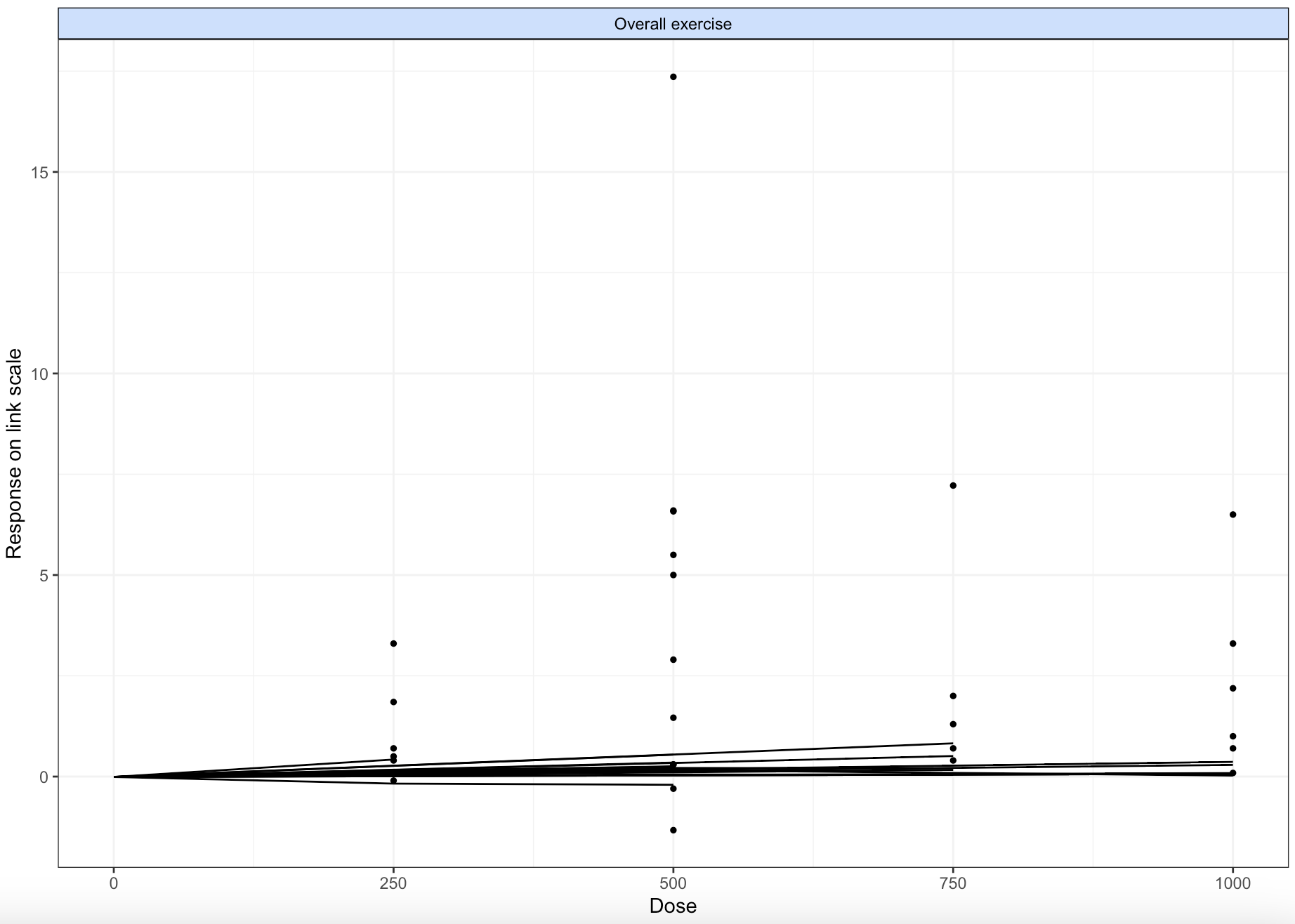


Figure S4. Dose-Response Patterns of Overall Exercise Effects on the Link Scale

Notes: The figure displays individual posterior response trajectories across standardized overall exercise dose levels on the link scale. Most trajectories demonstrated gradual dose-related changes, while a limited number of higher-response observations were observed at specific dose levels, reflecting variability in modeled responses. Overall, the patterns suggest a dose-associated trend with observable individual variation.
