# Supplementary material for: Optimal exercise modalities and dosages for improving depression in middle-aged and older adults with Parkinson's disease: A Bayesian Dose–response network meta-analysis
Source: PLoS One. 2026 Jul 23;21(7):e0354206. doi: 10.1371/journal.pone.0354206 (PMC13395444; doi:10.1371/journal.pone.0354206)
Supplement: S5 Fig — Panel-specific boxplots tracking modality-dependent median effect estimates, distributions, and variance metrics across cumulative dose levels. (DOCX) [file pone.0354206.s013.docx]

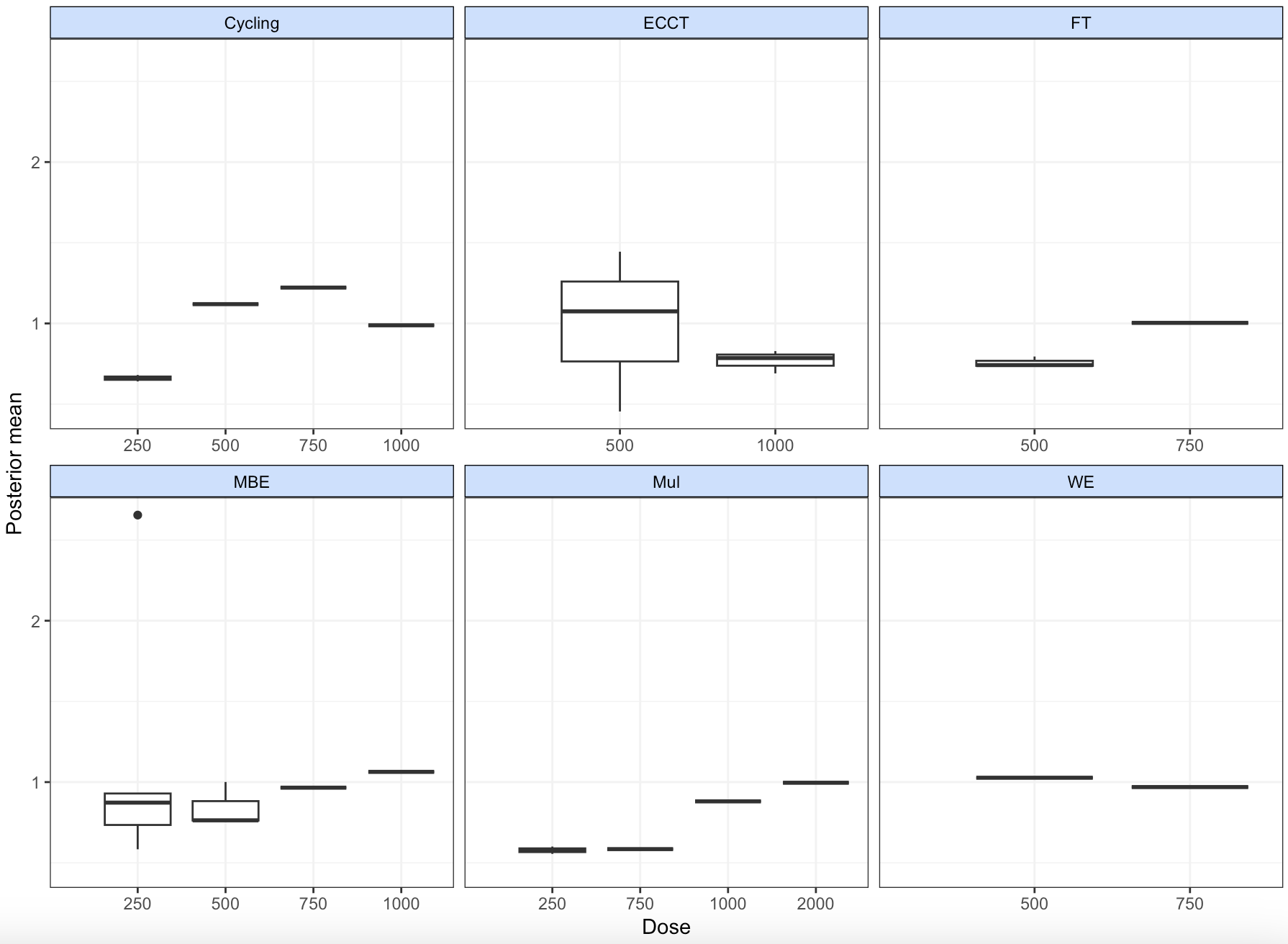


Figure S5. Distribution of Posterior Mean Effects Across Exercise Modalities and Dose Levels

Notes: Boxplots illustrate the distribution of posterior mean effect estimates across different exercise modalities and standardized dose levels. Each panel represents one exercise modality, with medians and interquartile ranges shown for each dose category and points indicating potential outliers. Overall, posterior mean estimates varied across modalities and dose levels, reflecting modality-specific dose–response distributions while maintaining generally consistent patterns within each modality.
