# Supplementary material for: Optimal exercise modalities and dosages for improving depression in middle-aged and older adults with Parkinson's disease: A Bayesian Dose–response network meta-analysis
Source: PLoS One. 2026 Jul 23;21(7):e0354206. doi: 10.1371/journal.pone.0354206 (PMC13395444; doi:10.1371/journal.pone.0354206)
Supplement: S6 Fig — Modality-stratified trajectory charts representing individual and modeled posterior response trends across cumulative standardized dose ranges. (DOCX) [file pone.0354206.s014.docx]

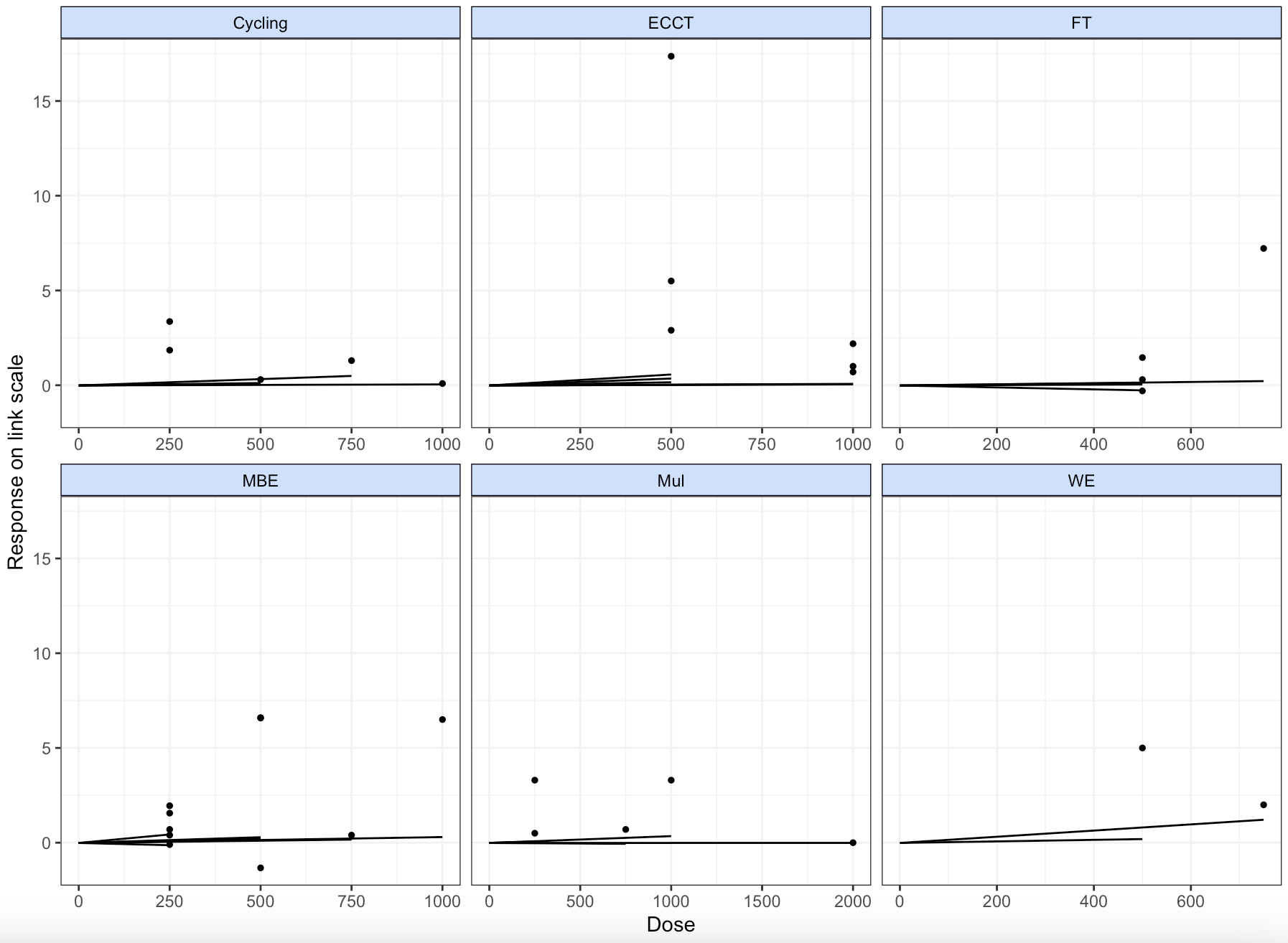


Figure S6. Dose–Response Patterns Across Exercise Modalities on the Link Scale

Notes: The figure presents individual response trajectories across standardized dose levels for each exercise modality on the link scale. Panels correspond to different exercise modalities, with lines illustrating modeled dose-related response patterns and points representing observed posterior responses. Across modalities, dose-associated changes were observed together with individual variability, indicating heterogeneous but coherent dose–response trends within each modality.
