# Supplementary material for: Optimal exercise modalities and dosages for improving depression in middle-aged and older adults with Parkinson's disease: A Bayesian Dose–response network meta-analysis
Source: PLoS One. 2026 Jul 23;21(7):e0354206. doi: 10.1371/journal.pone.0354206 (PMC13395444; doi:10.1371/journal.pone.0354206)
Supplement: Attachment — Submitted filename: Response to Reviewers.docx [file pone.0354206.s017.docx]

Dear Academic Editor and Reviewers,

We sincerely thank the Academic Editor and both reviewers for their careful evaluation of our manuscript and for the constructive comments. We appreciate the recognition of the value of this work and have revised the manuscript accordingly. The revisions mainly clarify the exercise intervention details, the quality assessment method, the use and limitations of MET-based dose standardisation, the interpretation of modality-specific and high-dose findings, and the limitations related to disease severity, medication status, intervention duration, and sparse evidence in some dose nodes. Below, we provide a point-by-point response.

Reviewer #1

Comment 1:
I think there is need to add more description about what type of exercise they are mentioning along with the dose.

Response 1:
Thank you very much for this helpful comment. We agree that exercise dose should be interpreted together with the specific exercise modality from which it was derived. In the revised manuscript, we clarified in the Methods and Results sections that exercise modalities were identified from the original intervention descriptions and linked with the corresponding MET values used for weekly dose calculation. We also revised the description of Supplementary Table 1 to indicate that it provides sample characteristics, exercise modalities, prescription parameters, assigned MET values, and weekly dose calculation details. In addition, we added a note below Supplementary Table 1 explaining that weekly exercise dose was calculated as MET-minutes/week using the formula: MET value × session duration × weekly frequency. These revisions make clearer that each dose estimate was based on specific exercise prescriptions rather than on dose alone.

Comment 2:
Also give some description about how they consider a score of 0-10 for quality assessment? Is there any earlier reports does the same?

Response 2:
Thank you for pointing this out. We have clarified the scoring method in the Quality Assessment section. Specifically, we now explain that the Physiotherapy Evidence Database scale contains 11 items, with the first item related to external validity and not included in the total score. The remaining 10 items contribute to the total score, with each item assigned 1 point when the criterion is clearly satisfied and 0 points when the criterion is not satisfied or not reported. Therefore, the total methodological quality score ranges from 0 to 10. We also clarified that this 0–10 scoring approach has been widely used in previous systematic reviews and meta-analyses of physiotherapy, rehabilitation, and exercise intervention trials. The interpretation of scores was also retained, with scores below 4 considered insufficient methodological quality, 4–5 indicating fair quality, 6–7 indicating moderate quality, and 8 or higher indicating high quality.

Reviewer #2

Comment 1:
The authors standardized exercise dose using the 2024 Adult and Older Adult Compendiums of Physical Activities. However, they must clarify whether these metabolic equivalents (METs) are truly representative of a Parkinson’s population.

Response 1:
Thank you for this important comment. We agree that MET values derived from general adult and older adult compendiums may not fully represent the actual physiological energy cost of exercise in individuals with Parkinson disease. In the revised manuscript, we clarified that, because no Parkinson disease-specific MET compendium is currently available, the 2024 Adult Compendium and Older Adult Compendium were used as standardised references to harmonise exercise dose across trials. We also added a limitation noting that motor symptoms, disease severity, functional status, and medication status may influence actual energy expenditure in Parkinson disease. Therefore, MET-minutes/week should be interpreted as a standardised relative dose metric for evidence synthesis rather than as a precise individual-level estimate of physiological energy expenditure.

Comment 2:
Authors should clarify why aerobic cycling and multicomponent training showed no stable benefit, which contradicts prior research. Specify if this is due to trial scarcity or a true lack of efficacy for depressive symptoms.

Response 2:
Thank you for this helpful suggestion. We have clarified this issue in the limitations section. We now state that the absence of stable significant dose ranges for aerobic cycling and multicomponent training should not be interpreted as definitive evidence of no efficacy for depressive symptoms. Rather, these findings may reflect sparse data within certain modality-dose nodes and heterogeneity in intervention content. This point is particularly relevant for multicomponent training, because programmes under this category may combine aerobic, resistance, balance, functional, cognitive, or behavioural components in different ways. Such variability may dilute a consistent modality-specific dose signal and reduce the stability of the estimated dose–response curves.

Comment 3:
Please distinguish whether the "instability" observed at 1000 MET-minutes reflects a biological saturation effect where physiological strain offsets mental health gains or simply a lack of sufficient data at high-dose intervals.

Response 3:
Thank you for raising this important interpretive issue. We have revised the limitations section to clarify that the instability observed at 1000 MET-minutes/week cannot be clearly distinguished as either a true biological saturation effect or an artefact of limited high-dose evidence. Given the sparse data and wider uncertainty at high-dose nodes, we now interpret this finding primarily as reduced statistical stability rather than as definitive evidence of a harmful or ineffective high-dose threshold. We also added that future trials with adequately represented high-dose exercise prescriptions are needed to distinguish biological saturation from data-driven uncertainty.

Comment 4:
The current model fails to account for disease stage, such as Hoehn and Yahr scale, or medication status. Given that dopaminergic function significantly influences both depression and the response to exercise, the authors should justify why these were not included as moderators.

Response 4:
Thank you for this important comment. We agree that disease severity and medication status are clinically relevant and may influence exercise response in Parkinson disease. In the revised limitations section, we clarified that subgroup heterogeneity across disease stage, cognitive status, functional capacity, and medication status was not modelled with fine granularity because these variables were not consistently or sufficiently reported across the included RCTs. Several studies did not provide extractable group-level data suitable for meta-regression. Including these factors as moderators would therefore have substantially reduced the analysable evidence base and increased model instability. We also added that future studies should report disease stage, medication status, baseline depressive symptom severity, and individual functional profiles in a standardised manner to support more clinically stratified dose–response modelling.

Comment 5:
Please justify the four-week minimum duration for inclusion. The authors should clarify if the length of the intervention influenced the dose-response curve, as neuroplastic changes typically require more sustained periods to manifest.

Response 5:
Thank you for this helpful comment. We revised the Eligibility Criteria section to clarify the rationale for the four-week minimum duration. This threshold was used to focus on sustained exercise interventions rather than acute or very short-term exposure studies, and to ensure that the included interventions represented repeated training exposure with sufficient prescription stability for weekly dose estimation. We also acknowledged in the limitations section that intervention duration may have influenced the estimated dose–response curves. Although the four-week criterion reduced the inclusion of acute or very short-term studies, intervention length still varied across trials, and the current evidence base did not allow intervention duration to be modelled with sufficient precision as an independent moderator. Therefore, the estimated dose–response relationship should be interpreted as reflecting weekly exercise dose within sustained interventions, rather than as fully separating the effects of weekly dose from total intervention length.

Comment 6:
Please explain the finding that walking only becomes significant at high doses (≥670 MET-min). This suggests that low-to-moderate walking is ineffective for depression in this population, which contradicts standard clinical recommendations and warrants further discussion.

Response 6:
Thank you for this valuable comment. We have revised the limitations section to clarify that the finding for walking should be interpreted cautiously. Although walking reached statistical significance only at higher dose nodes of 670 MET-minutes/week or above, this should not be interpreted as evidence that low-to-moderate dose walking is ineffective. The finding may reflect limited trial representation, uneven dose distribution, and wider uncertainty around walking-specific estimates at lower dose levels. We also clarified that walking remains a clinically accessible and commonly recommended exercise option, but in the present dose–response model for depressive symptoms in Parkinson disease, a clearer statistical signal was observed only at higher weekly doses. Future studies are needed to clarify the dose range over which walking exercise may provide stable antidepressant benefits.

Comment 7:
To further enhance the Related Work and Future Directions sections, I recommend incorporating the following three papers to broaden the scholarly impact and technical depth of the manuscript:
Tang et al. (2024); Abrar et al. (2026); Twala (2025).

Response 7:
Thank you for recommending these recent publications. We carefully reviewed the suggested studies and incorporated the relevant evidence while maintaining the focus of the manuscript on exercise dose and dose–response modelling. The study by Tang et al. was added to the discussion because it directly addresses optimal exercise dose and modality for depressive symptoms in older adults and helps contextualise our findings within broader geriatric depression evidence. We also considered the two AI-related publications. However, because the present manuscript is focused on exercise modality and dose–response relationships rather than artificial intelligence or assistive technology, we did not expand the main discussion toward AI-based diagnosis or intervention. Instead, we revised the Future Directions section to remain centred on multidimensional dose–response modelling, including weekly dose, accumulated dose, intervention duration, modality characteristics, and clinical phenotypes. This revision allows the manuscript to address the reviewer’s suggestion while preserving the central dose-focused scope of the study.

We again thank the Academic Editor and reviewers for their constructive comments. We believe these revisions have improved the clarity, methodological transparency, and interpretive caution of the manuscript.
