# Supplementary material for: Optimal exercise modalities and dosages for improving depression in middle-aged and older adults with Parkinson's disease: A Bayesian Dose–response network meta-analysis
Source: PLoS One. 2026 Jul 23;21(7):e0354206. doi: 10.1371/journal.pone.0354206 (PMC13395444; doi:10.1371/journal.pone.0354206)
Supplement: Peer review history

# Peer review: Decision Letter 0

**DOI:** 10.1371/journal.pone.0354206.r001

**Authors:** Bala Munipalli

8 Jun 2026

PONE-D-26-10375

Optimal Exercise Modalities and Dosages for Improving Depression in Middle-Aged and Older Adults with Parkinson's Disease: A Bayesian Dose–Response Network Meta-Analysis

PLOS One

Dear Dr. Zeng,

Thank you for submitting your manuscript to PLOS ONE. After careful consideration, we feel that it has merit but does not fully meet PLOS ONE’s publication criteria as it currently stands. Therefore, we invite you to submit a revised version of the manuscript that addresses the points raised during the review process.

==============================

- Please provide additional details on the exercise interventions, including the specific types of exercise evaluated in each dose category, rather than reporting exercise dose alone.
- Clarify the methodology used for the 0–10 quality assessment score, including the scoring criteria, interpretation of scores, and whether this approach has been adopted in previous studies. Appropriate references should be provided.
- Please justify the use of MET values derived from the 2024 Adult and Older Adult Compendiums and discuss their applicability to individuals with Parkinson’s disease.
- Further discuss findings that differ from previous evidence, particularly the lack of stable benefits observed for aerobic cycling and multicomponent training, and the apparent requirement for higher-dose walking interventions to achieve significant improvements in depressive symptoms.
- Clarify whether the instability observed at higher exercise doses reflects a biological saturation effect, limited data availability, or another methodological consideration.
- Discuss the potential influence of disease severity and medication status on the observed outcomes and justify why these factors were not included as moderators in the analysis.
- Provide a rationale for the minimum intervention duration of four weeks and discuss whether intervention length may have influenced the dose–response relationship.
- Consider expanding the discussion by incorporating recent relevant literature and further acknowledge limitations related to intervention heterogeneity, sparse high-dose data, and potential unmeasured confounding factors.

==============================

Please submit your revised manuscript by Jul 23 2026 11:59PM. If you will need more time than this to complete your revisions, please reply to this message or contact the journal office at plosone@plos.org. When you're ready to submit your revision, log on to https://www.editorialmanager.com/pone/ and select the 'Submissions Needing Revision' folder to locate your manuscript file.

Please include the following items when submitting your revised manuscript:

- A letter that responds to each point raised by the academic editor and reviewer(s). You should upload this letter as a separate file labeled 'Response to Reviewers'.
- A marked-up copy of your manuscript that highlights changes made to the original version. You should upload this as a separate file labeled 'Revised Manuscript with Track Changes'.
- A revised rmanuscript without tracked changes. You should upload this as a separate file labeled 'Manuscript'.

If you would like to make changes to your financial disclosure, please include your updated statement in your cover letter. Guidelines for resubmitting your figure files are available below the reviewer comments at the end of this letter.

If applicable, we recommend that you deposit your laboratory protocols in protocols.io to enhance the reproducibility of your results. Protocols.io assigns your protocol its own identifier (DOI) so that it can be cited independently in the future. For instructions see: https://journals.plos.org/plosone/s/submission-guidelines#loc-laboratory-protocols. Additionally, PLOS ONE offers an option for publishing peer-reviewed Lab Protocol articles, which describe protocols hosted on protocols.io. Read more information on sharing protocols at https://plos.org/protocols?utm_medium=editorial-email&utm_source=authorletters&utm_campaign=protocols.

As the corresponding author, your ORCID iD is verified in the submission system and will appear in the published article. PLOS supports the use of ORCID, and we encourage all coauthors to register for an ORCID iD and use it as well. Please encourage your coauthors to verify their ORCID iD within the submission system before final acceptance, as unverified ORCID iDs will not appear in the published article. *Only*  the individual author can complete the verification step; PLOS staff *cannot*  verify ORCID iDs on behalf of authors.

We look forward to receiving your revised manuscript.

Kind regards,

Bala Munipalli

Academic Editor

PLOS One

Journal Requirements:

When submitting your revision, we need you to address these additional requirements.

1. Please ensure that your manuscript meets PLOS ONE's style requirements, including those for file naming. The PLOS ONE style templates can be found at

https://journals.plos.org/plosone/s/file?id=wjVg/PLOSOne_formatting_sample_main_body.pdf and

https://journals.plos.org/plosone/s/file?id=ba62/PLOSOne_formatting_sample_title_authors_affiliations.pdf

2. We suggest you thoroughly copyedit your manuscript for language usage, spelling, and grammar. If you do not know anyone who can help you do this, you may wish to consider employing a professional scientific editing service.

The American Journal Experts (AJE) (https://www.aje.com/) is one such service that has extensive experience helping authors meet PLOS guidelines and can provide language editing, translation, manuscript formatting, and figure formatting to ensure your manuscript meets our submission guidelines. Please note that having the manuscript copyedited by AJE or any other editing services does not guarantee selection for peer review or acceptance for publication.

Upon resubmission, please provide the following:

The name of the colleague or the details of the professional service that edited your manuscript

A copy of your manuscript showing your changes by either highlighting them or using track changes (uploaded as a *supporting information* file)

A clean copy of the edited manuscript (uploaded as the new *manuscript* file)

3. PLOS requires an ORCID iD for the corresponding author in Editorial Manager on papers submitted after December 6th, 2016. Please ensure that you have an ORCID iD and that it is validated in Editorial Manager. To do this, go to ‘Update my Information’ (in the upper left-hand corner of the main menu), and click on the Fetch/Validate link next to the ORCID field. This will take you to the ORCID site and allow you to create a new iD or authenticate a pre-existing iD in Editorial Manager.

4. Please include captions for your Supporting Information files at the end of your manuscript, and update any in-text citations to match accordingly. Please see our Supporting Information guidelines for more information: http://journals.plos.org/plosone/s/supporting-information.

5. If the reviewer comments include a recommendation to cite specific previously published works, please review and evaluate these publications to determine whether they are relevant and should be cited. There is no requirement to cite these works unless the editor has indicated otherwise.

6. Please review your reference list to ensure that it is complete and correct. If you have cited papers that have been retracted, please include the rationale for doing so in the manuscript text, or remove these references and replace them with relevant current references. Any changes to the reference list should be mentioned in the rebuttal letter that accompanies your revised manuscript. If you need to cite a retracted article, indicate the article’s retracted status in the References list and also include a citation and full reference for the retraction notice.

[Note: HTML markup is below. Please do not edit.]

Reviewers' comments:

Reviewer's Responses to Questions

**Comments to the Author**

1. Is the manuscript technically sound, and do the data support the conclusions?

The manuscript must describe a technically sound piece of scientific research with data that supports the conclusions. Experiments must have been conducted rigorously, with appropriate controls, replication, and sample sizes. The conclusions must be drawn appropriately based on the data presented.

Reviewer #1: Partly

Reviewer #2: Yes

**********

2. Has the statistical analysis been performed appropriately and rigorously?

Reviewer #1: N/A

Reviewer #2: Yes

**********

3. Have the authors made all data underlying the findings in their manuscript fully available?

The PLOS Data policy requires authors to make all data underlying the findings described in their manuscript fully available without restriction, with rare exception (please refer to the Data Availability Statement in the manuscript PDF file). The data should be provided as part of the manuscript or its supporting information, or deposited to a public repository. For example, in addition to summary statistics, the data points behind means, medians and variance measures should be available. If there are restrictions on publicly sharing data—e.g. participant privacy or use of data from a third party—those must be specified.

Reviewer #1: Yes

Reviewer #2: Yes

**********

4. Is the manuscript presented in an intelligible fashion and written in standard English?

PLOS ONE does not copyedit accepted manuscripts, so the language in submitted articles must be clear, correct, and unambiguous. Any typographical or grammatical errors should be corrected at revision, so please note any specific errors here.

Reviewer #1: Yes

Reviewer #2: Yes

**********

5. Review Comments to the Author

Please use the space provided to explain your answers to the questions above. You may also include additional comments for the author, including concerns about dual publication, research ethics, or publication ethics. (Please upload your review as an attachment if it exceeds 20,000 characters)

Reviewer #1: I think there is need to add more description about what type of exercise they are mentioning along with the dose?

also give some description about how they consider a score of 0-10 for quality assessment? is there any earlier reports does the same?

Reviewer #2: This research study utilizes a Bayesian network meta-analysis to investigate the relationship between exercise dosage and depressive symptoms in older adults with Parkinson’s disease. The authors analyzed twenty-five randomized controlled trials to determine that exercise provides a non-linear benefit, meaning that improvements peak at a specific intensity rather than increasing indefinitely. The data identifies an optimal range of 440 to 670 MET-minutes per week, with the most significant relief occurring at approximately 560 MET-minutes. To further enhance the manuscript’s clarity, impact, and scholarly rigor, I recommend the following minor revisions:

1- The authors standardized exercise dose using the 2024 Adult and Older Adult Compendiums of Physical Activities. However, they must clarify whether these metabolic equivalents (METs) are truly representative of a Parkinson’s population.

2- Authors should clarify why aerobic cycling and multicomponent training showed no stable benefit, which contradicts prior research. Specify if this is due to trial scarcity or a true lack of efficacy for depressive symptoms.

3- Please distinguish whether the "instability" observed at 1000 MET-minutes reflects a biological saturation effect where physiological strain offsets mental health gains or simply a lack of sufficient data at high-dose intervals.

4- The current model fails to account for disease stage (Hoehn and Yahr scale) or medication status. Given that dopaminergic function significantly influences both depression and the response to exercise, the authors should justify why these were not included as moderators.

5- Please justify the four-week minimum duration for inclusion. The authors should clarify if the length of the intervention influenced the dose-response curve, as neuroplastic changes typically require more sustained periods to manifest

6- Please explain the finding that walking only becomes significant at high doses (≥670 MET-min). This suggests that low-to-moderate walking is ineffective for depression in this population, which contradicts standard clinical recommendations and warrants further discussion.

7- To further enhance the Related Work and Future Directions sections, I recommend incorporating the following three papers to broaden the scholarly impact and technical depth of the manuscript:

• Tang, L., Zhang, L., Liu, Y., Li, Y., Yang, L., Zou, M., ... & Li, Z. (2024). Optimal dose and type of exercise to improve depressive symptoms in older adults: a systematic review and network meta-analysis. BMC geriatrics, 24(1), 505.

• Abrar, M., Ur Rehman, M., Khalid, S., & Ullah, R. (2026). The intersection of artificial intelligence and assistive technologies in the diagnosis and intervention of mental health conditions. Artificial Intelligence Review, 59(2).

• Twala, B. (2025). AI-driven precision diagnosis and treatment in Parkinson’s disease: a comprehensive review and experimental analysis. Frontiers in Aging Neuroscience, 17, 1638340.

**********

6. PLOS authors have the option to publish the peer review history of their article (what does this mean?). If published, this will include your full peer review and any attached files.

If you choose “no”, your identity will remain anonymous but your review may still be made public.

**Do you want your identity to be public for this peer review?**  For information about this choice, including consent withdrawal, please see our Privacy Policy.

Reviewer #1: No

Reviewer #2: No

**********

[NOTE: If reviewer comments were submitted as an attachment file, they will be attached to this email and accessible via the submission site. Please log into your account, locate the manuscript record, and check for the action link "View Attachments". If this link does not appear, there are no attachment files.]

To ensure your figures meet our technical requirements, please review our figure guidelines: https://journals.plos.org/plosone/s/figures

You may also use PLOS’s free figure tool, NAAS, to help you prepare publication quality figures: https://journals.plos.org/plosone/s/figures#loc-tools-for-figure-preparation.

NAAS will assess whether your figures meet our technical requirements by comparing each figure against our figure specifications.

# Author comment: Author response to Decision Letter 1

**DOI:** 10.1371/journal.pone.0354206.r002

10 Jun 2026

Dear Academic Editor and Reviewers,

We sincerely thank the Academic Editor and both reviewers for their careful evaluation of our manuscript and for the constructive comments. We appreciate the recognition of the value of this work and have revised the manuscript accordingly. The revisions mainly clarify the exercise intervention details, the quality assessment method, the use and limitations of MET-based dose standardisation, the interpretation of modality-specific and high-dose findings, and the limitations related to disease severity, medication status, intervention duration, and sparse evidence in some dose nodes. Below, we provide a point-by-point response.

Reviewer #1

Comment 1:

I think there is need to add more description about what type of exercise they are mentioning along with the dose.

Response 1:

Thank you very much for this helpful comment. We agree that exercise dose should be interpreted together with the specific exercise modality from which it was derived. In the revised manuscript, we clarified in the Methods and Results sections that exercise modalities were identified from the original intervention descriptions and linked with the corresponding MET values used for weekly dose calculation. We also revised the description of Supplementary Table 1 to indicate that it provides sample characteristics, exercise modalities, prescription parameters, assigned MET values, and weekly dose calculation details. In addition, we added a note below Supplementary Table 1 explaining that weekly exercise dose was calculated as MET-minutes/week using the formula: MET value × session duration × weekly frequency. These revisions make clearer that each dose estimate was based on specific exercise prescriptions rather than on dose alone.

Comment 2:

Also give some description about how they consider a score of 0-10 for quality assessment? Is there any earlier reports does the same?

Response 2:

Thank you for pointing this out. We have clarified the scoring method in the Quality Assessment section. Specifically, we now explain that the Physiotherapy Evidence Database scale contains 11 items, with the first item related to external validity and not included in the total score. The remaining 10 items contribute to the total score, with each item assigned 1 point when the criterion is clearly satisfied and 0 points when the criterion is not satisfied or not reported. Therefore, the total methodological quality score ranges from 0 to 10. We also clarified that this 0–10 scoring approach has been widely used in previous systematic reviews and meta-analyses of physiotherapy, rehabilitation, and exercise intervention trials. The interpretation of scores was also retained, with scores below 4 considered insufficient methodological quality, 4–5 indicating fair quality, 6–7 indicating moderate quality, and 8 or higher indicating high quality.

Reviewer #2

Comment 1:

The authors standardized exercise dose using the 2024 Adult and Older Adult Compendiums of Physical Activities. However, they must clarify whether these metabolic equivalents (METs) are truly representative of a Parkinson’s population.

Response 1:

Thank you for this important comment. We agree that MET values derived from general adult and older adult compendiums may not fully represent the actual physiological energy cost of exercise in individuals with Parkinson disease. In the revised manuscript, we clarified that, because no Parkinson disease-specific MET compendium is currently available, the 2024 Adult Compendium and Older Adult Compendium were used as standardised references to harmonise exercise dose across trials. We also added a limitation noting that motor symptoms, disease severity, functional status, and medication status may influence actual energy expenditure in Parkinson disease. Therefore, MET-minutes/week should be interpreted as a standardised relative dose metric for evidence synthesis rather than as a precise individual-level estimate of physiological energy expenditure.

Comment 2:

Authors should clarify why aerobic cycling and multicomponent training showed no stable benefit, which contradicts prior research. Specify if this is due to trial scarcity or a true lack of efficacy for depressive symptoms.

Response 2:

Thank you for this helpful suggestion. We have clarified this issue in the limitations section. We now state that the absence of stable significant dose ranges for aerobic cycling and multicomponent training should not be interpreted as definitive evidence of no efficacy for depressive symptoms. Rather, these findings may reflect sparse data within certain modality-dose nodes and heterogeneity in intervention content. This point is particularly relevant for multicomponent training, because programmes under this category may combine aerobic, resistance, balance, functional, cognitive, or behavioural components in different ways. Such variability may dilute a consistent modality-specific dose signal and reduce the stability of the estimated dose–response curves.

Comment 3:

Please distinguish whether the "instability" observed at 1000 MET-minutes reflects a biological saturation effect where physiological strain offsets mental health gains or simply a lack of sufficient data at high-dose intervals.

Response 3:

Thank you for raising this important interpretive issue. We have revised the limitations section to clarify that the instability observed at 1000 MET-minutes/week cannot be clearly distinguished as either a true biological saturation effect or an artefact of limited high-dose evidence. Given the sparse data and wider uncertainty at high-dose nodes, we now interpret this finding primarily as reduced statistical stability rather than as definitive evidence of a harmful or ineffective high-dose threshold. We also added that future trials with adequately represented high-dose exercise prescriptions are needed to distinguish biological saturation from data-driven uncertainty.

Comment 4:

The current model fails to account for disease stage, such as Hoehn and Yahr scale, or medication status. Given that dopaminergic function significantly influences both depression and the response to exercise, the authors should justify why these were not included as moderators.

Response 4:

Thank you for this important comment. We agree that disease severity and medication status are clinically relevant and may influence exercise response in Parkinson disease. In the revised limitations section, we clarified that subgroup heterogeneity across disease stage, cognitive status, functional capacity, and medication status was not modelled with fine granularity because these variables were not consistently or sufficiently reported across the included RCTs. Several studies did not provide extractable group-level data suitable for meta-regression. Including these factors as moderators would therefore have substantially reduced the analysable evidence base and increased model instability. We also added that future studies should report disease stage, medication status, baseline depressive symptom severity, and individual functional profiles in a standardised manner to support more clinically stratified dose–response modelling.

Comment 5:

Please justify the four-week minimum duration for inclusion. The authors should clarify if the length of the intervention influenced the dose-response curve, as neuroplastic changes typically require more sustained periods to manifest.

Response 5:

Thank you for this helpful comment. We revised the Eligibility Criteria section to clarify the rationale for the four-week minimum duration. This threshold was used to focus on sustained exercise interventions rather than acute or very short-term exposure studies, and to ensure that the included interventions represented repeated training exposure with sufficient prescription stability for weekly dose estimation. We also acknowledged in the limitations section that intervention duration may have influenced the estimated dose–response curves. Although the four-week criterion reduced the inclusion of acute or very short-term studies, intervention length still varied across trials, and the current evidence base did not allow intervention duration to be modelled with sufficient precision as an independent moderator. Therefore, the estimated dose–response relationship should be interpreted as reflecting weekly exercise dose within sustained interventions, rather than as fully separating the effects of weekly dose from total intervention length.

Comment 6:

Please explain the finding that walking only becomes significant at high doses (≥670 MET-min). This suggests that low-to-moderate walking is ineffective for depression in this population, which contradicts standard clinical recommendations and warrants further discussion.

Response 6:

Thank you for this valuable comment. We have revised the limitations section to clarify that the finding for walking should be interpreted cautiously. Although walking reached statistical significance only at higher dose nodes of 670 MET-minutes/week or above, this should not be interpreted as evidence that low-to-moderate dose walking is ineffective. The finding may reflect limited trial representation, uneven dose distribution, and wider uncertainty around walking-specific estimates at lower dose levels. We also clarified that walking remains a clinically accessible and commonly recommended exercise option, but in the present dose–response model for depressive symptoms in Parkinson disease, a clearer statistical signal was observed only at higher weekly doses. Future studies are needed to clarify the dose range over which walking exercise may provide stable antidepressant benefits.

Comment 7:

To further enhance the Related Work and Future Directions sections, I recommend incorporating the following three papers to broaden the scholarly impact and technical depth of the manuscript:

Tang et al. (2024); Abrar et al. (2026); Twala (2025).

Response 7:

Thank you for recommending these recent publications. We carefully reviewed the suggested studies and incorporated the relevant evidence while maintaining the focus of the manuscript on exercise dose and dose–response modelling. The study by Tang et al. was added to the discussion because it directly addresses optimal exercise dose and modality for depressive symptoms in older adults and helps contextualise our findings within broader geriatric depression evidence. We also considered the two AI-related publications. However, because the present manuscript is focused on exercise modality and dose–response relationships rather than artificial intelligence or assistive technology, we did not expand the main discussion toward AI-based diagnosis or intervention. Instead, we revised the Future Directions section to remain centred on multidimensional dose–response modelling, including weekly dose, accumulated dose, intervention duration, modality characteristics, and clinical phenotypes. This revision allows the manuscript to address the reviewer’s suggestion while preserving the central dose-focused scope of the study.

We again thank the Academic Editor and reviewers for their constructive comments. We believe these revisions have improved the clarity, methodological transparency, and interpretive caution of the manuscript.

Attachment Submitted filename: Response to Reviewers.docx

# Peer review: Decision Letter 1

**DOI:** 10.1371/journal.pone.0354206.r003

**Authors:** Bala Munipalli

6 Jul 2026

Optimal Exercise Modalities and Dosages for Improving Depression in Middle-Aged and Older Adults with Parkinson's Disease: A Bayesian Dose–Response Network Meta-Analysis

PONE-D-26-10375R1

Dear Dr. Zeng,

We’re pleased to inform you that your manuscript has been judged scientifically suitable for publication and will be formally accepted for publication once it meets all outstanding technical requirements.The manuscript has been substantially improved in response to the previous round of review. One remaining point concerns the interpretation of the proposed mechanistic framework. Before the manuscript can be accepted, please revise the Discussion to clarify that the observed improvements in depressive symptoms provide clinical evidence of efficacy but do not constitute direct mechanistic evidence. The discussion of prefrontal regulation and cortico-striatal network engagement should be presented as a hypothesis informed by prior literature, and the limitations section should note that future studies incorporating multimodal mechanistic measures are needed to validate these proposed pathways

Within one week, you’ll receive an e-mail detailing the required amendments. When these have been addressed, you’ll receive a formal acceptance letter and your manuscript will be scheduled for publication.

An invoice will be generated when your article is formally accepted. Please note, if your institution has a publishing partnership with PLOS and your article meets the relevant criteria, all or part of your publication costs will be covered. Please make sure your user information is up-to-date by logging into Editorial Manager at Editorial Manager® and clicking the ‘Update My Information' link at the top of the page. For questions related to billing, please contact billing support.

If your institution or institutions have a press office, please notify them about your upcoming paper to help maximize its impact. If they’ll be preparing press materials, please inform our press team as soon as possible -- no later than 48 hours after receiving the formal acceptance. Your manuscript will remain under strict press embargo until 2 pm Eastern Time on the date of publication. For more information, please contact onepress@plos.org.

Kind regards,

Bala Munipalli

Academic Editor

PLOS One

Additional Editor Comments (optional):

Reviewers' comments:

Reviewer's Responses to Questions

**Comments to the Author**

1. If the authors have adequately addressed your comments raised in a previous round of review and you feel that this manuscript is now acceptable for publication, you may indicate that here to bypass the “Comments to the Author” section, enter your conflict of interest statement in the “Confidential to Editor” section, and submit your "Accept" recommendation.

Reviewer #1: (No Response)

Reviewer #2: All comments have been addressed

**********

2. Is the manuscript technically sound, and do the data support the conclusions?

The manuscript must describe a technically sound piece of scientific research with data that supports the conclusions. Experiments must have been conducted rigorously, with appropriate controls, replication, and sample sizes. The conclusions must be drawn appropriately based on the data presented.

Reviewer #1: Yes

Reviewer #2: Yes

**********

3. Has the statistical analysis been performed appropriately and rigorously?

Reviewer #1: I Don't Know

Reviewer #2: Yes

**********

4. Have the authors made all data underlying the findings in their manuscript fully available?

The PLOS Data policy requires authors to make all data underlying the findings described in their manuscript fully available without restriction, with rare exception (please refer to the Data Availability Statement in the manuscript PDF file). The data should be provided as part of the manuscript or its supporting information, or deposited to a public repository. For example, in addition to summary statistics, the data points behind means, medians and variance measures should be available. If there are restrictions on publicly sharing data—e.g. participant privacy or use of data from a third party—those must be specified.

Reviewer #1: Yes

Reviewer #2: Yes

**********

5. Is the manuscript presented in an intelligible fashion and written in standard English?

PLOS ONE does not copyedit accepted manuscripts, so the language in submitted articles must be clear, correct, and unambiguous. Any typographical or grammatical errors should be corrected at revision, so please note any specific errors here.

Reviewer #1: Yes

Reviewer #2: (No Response)

**********

6. Review Comments to the Author

Please use the space provided to explain your answers to the questions above. You may also include additional comments for the author, including concerns about dual publication, research ethics, or publication ethics. (Please upload your review as an attachment if it exceeds 20,000 characters)

Reviewer #1: The study used depressive symptom scores as the primary outcome, which capture clinically relevant changes but do not provide direct mechanistic evidence. Neuroimaging markers, biological measures, and indices of dopaminergic function were not integrated. Consequently, the study does not establish a direct chain linking dose, mechanistic indicators, and improvement in depressive symptoms, despite proposing a framework involving prefrontal regulation and cortico-striatal network engagement. Further work should incorporate multimodal mechanistic measures within dose prediction models and utilize longitudinal designs to move from behavioral inference to mechanistic validation.

Reviewer #2: (No Response)

**********

7. PLOS authors have the option to publish the peer review history of their article (what does this mean?). If published, this will include your full peer review and any attached files.

If you choose “no”, your identity will remain anonymous but your review may still be made public.

**Do you want your identity to be public for this peer review?**  For information about this choice, including consent withdrawal, please see our Privacy Policy.

Reviewer #1: No

Reviewer #2: No

**********

# Editor report: Acceptance letter

**DOI:** 10.1371/journal.pone.0354206.r004

**Authors:** Bala Munipalli

PONE-D-26-10375R1

PLOS One

Dear Dr. Zeng,

I'm pleased to inform you that your manuscript has been deemed suitable for publication in PLOS One. Congratulations! Your manuscript is now being handed over to our production team.

At this stage, our production department will prepare your paper for publication. This includes ensuring the following:

* All references, tables, and figures are properly cited

* All relevant supporting information is included in the manuscript submission,

* There are no issues that prevent the paper from being properly typeset

You will receive further instructions from the production team, including instructions on how to review your proof when it is ready. Please keep in mind that we are working through a large volume of accepted articles, so please give us a few days to review your paper and let you know the next and final steps.

Lastly, if your institution or institutions have a press office, please let them know about your upcoming paper now to help maximize its impact. If they'll be preparing press materials, please inform our press team within the next 48 hours. Your manuscript will remain under strict press embargo until 2 pm Eastern Time on the date of publication. For more information, please contact onepress@plos.org.

You will receive an invoice from PLOS for your publication fee after your manuscript has reached the completed accept phase. If you receive an email requesting payment before acceptance or for any other service, this may be a phishing scheme. Learn how to identify phishing emails and protect your accounts at https://explore.plos.org/phishing.

If we can help with anything else, please email us at customercare@plos.org.

Thank you for submitting your work to PLOS ONE and supporting open access.

Kind regards,

PLOS ONE Editorial Office Staff

on behalf of

Dr. Bala Munipalli

Academic Editor

PLOS One
